# Screening and Analysis of *Anaplasma marginale* Tunisian Isolates Reveal the Diversity of *lipA* Phylogeographic Marker and the Conservation of OmpA Protein Vaccine Candidate

**DOI:** 10.3389/fvets.2021.731200

**Published:** 2021-10-21

**Authors:** Hanène Belkahia, Meriem Ben Abdallah, Rihab Andolsi, Rachid Selmi, Sayed Zamiti, Myriam Kratou, Moez Mhadhbi, Mohamed Aziz Darghouth, Lilia Messadi, Mourad Ben Said

**Affiliations:** ^1^Service de Microbiologie et Immunologie, Ecole Nationale de Médecine Vétérinaire, University of Manouba, Sidi Thabet, Tunisia; ^2^Ministère de la Défense Nationale, Direction Générale de la Santé Militaire, Service Vétérinaire, Tunis, Tunisia; ^3^Service de Parasitologie, Ecole Nationale de Médecine Vétérinaire, University of Manouba, Sidi Thabet, Tunisia; ^4^Département des Sciences Fondamentales, Institut Supérieur de Biotechnologie de Sidi Thabet, University of Manouba, Sidi Thabet, Tunisia

**Keywords:** *Anaplasma marginale*, *lipA* and *sucB* markers, phylogeographic analysis, OmpA vaccine candidate, conservation assessment, Tunisia

## Abstract

Bovine anaplasmosis caused by *Anaplasma marginale* is a disease responsible for serious animal health problems and great economic losses all over the world. Thereby, the identification of *A. marginale* isolates from various bioclimatic areas in each country, the phylogeographic analysis of these isolates based on the most informative markers, and the evaluation of the most promising candidate antigens are crucial steps in developing effective vaccines against a wide range of *A. marginale* strains. In order to contribute to this challenge, a total of 791 bovine samples from various bioclimatic areas of Tunisia were tested for the occurrence of *A. marginale* DNA through *msp4* gene fragment amplification. Phylogeographic analysis was performed by using *lipA* and *sucB* gene analyses, and the genetic relationship with previously characterized *A. marginale* isolates and strains was analyzed by applying similarity comparison and phylogenetic analysis. To evaluate the conservation of OmpA protein vaccine candidate, almost complete *ompA* nucleotide sequences were also obtained from Tunisian isolates, and various bioinformatics software were used in order to analyze the physicochemical properties and the secondary and tertiary structures of their deduced proteins and to predict their immunodominant epitopes of B and T cells. *A. marginale* DNA was detected in 19 bovine samples (2.4%). Risk factor analysis shows that cattle derived from subhumid bioclimatic area were more infected than those that originated from other areas. The analysis of *lipA* phylogeographic marker indicated a higher diversity of Tunisian *A. marginale* isolates compared with other available worldwide isolates and strains. Molecular, phylogenetic, and immuno-informatics analyses of the vaccine candidate OmpA protein demonstrated that this antigen and its predicted immunodominant epitopes of B and T cells appear to be highly conserved between Tunisian isolates and compared with isolates from other countries, suggesting that the minimal intraspecific modifications will not affect the potential cross-protective capacity of humoral and cell-mediated immune responses against multiple *A. marginale* worldwide strains.

## Introduction

Bovine anaplasmosis caused by *Anaplasma marginale* is a disease responsible for serious animal health problems and great economic losses worldwide (1). *A. marginale*, a species of Gram-negative intracellular obligate bacteria, is the most pathogenic agent responsible for this disease (2). It commonly infects erythrocytes of cattle and wild ruminants and is considered among the most prevalent bacterium transmitted by ticks in the world (3). The major clinical signs related to this disease are hemolytic anemia, jaundice, fever, loss of weight, and decreased milk production. Sometimes, abortion and death of infected animals may be observed in chronic form (3).

Despite the economic impact of this disease, no commercial vaccines against bovine anaplasmosis were developed until now, and only immuno-antigenic elements of the outer membrane preparation including the major surface proteins are studied (1, 4, 5). However, these protein candidates did not confer sufficient immune protection (4, 6, 7). Recently, adhesins were considered as particularly interesting vaccine candidates given that they have an essential role in the survival of obligate intracellular bacteria and are more conserved than other candidate vaccine proteins (8). Among them, OmpA, annotated Am854 in the genome of *A. marginale*, has been classified as an adhesin, which has an important function in the entrance of *A. marginale* in vertebrate host and arthropod vector cells (9). Therefore, this protein could be considered as one of the highly promising vaccine candidates (8).

However, knowledge of the phylogeographic relationships between isolates of *A. marginale* is crucial to better prevent and control infections throughout the world. Indeed, it contributes to the discovery of the mechanisms involved in the difference of pathogenicity of *A. marginale* by passing from one isolate to another (10). Nonetheless, some genes like *msp4* and *msp1a* previously used for *A. marginale* genotyping are relatively efficient for isolate discrimination at the regional level; however, they are not interesting markers for analyzing the phylogeographic relationships between worldwide isolates and strains (11–13).

Therefore, a first multi-locus sequence typing (MLST) scheme for *A. marginale* was developed by Guillemi et al. (10) on 58 isolates and strains from different regions of the world to investigate whether geographically close isolates will have similar sequence types (STs) to each other than will geographically more distant isolates and strains. A total of seven loci (*dnaA, ftsZ, groEL, lipA, secY, recA*, and *sucB*) were amplified and sequenced, and different STs were obtained on the basis of the nucleotide diversity of the concatenated fragment. However, the authors did not find a clear relationship between geographic regions and STs isolated from various worldwide isolates and strains (10).

More recently, Ben Said et al. (14) examined independently each earlier cited housekeeping gene locus initially employed in the MLST scheme by using the single gene analysis (SGA). This method was performed in order to search a possible phylogeographic resolution at least for one in each of the seven genes. The phylogenetic analysis of each marker revealed that, out of the seven analyzed genes, two (*lipA* and *sucB*) were found to be interesting phylogeographically and allowed the classification of Tunisian isolates and those found in GenBank according to the continents (*lipA*) and according to the New and Old World (*sucB*) (14).

In Tunisia, cross-sectional and longitudinal surveys on bovine anaplasmosis using molecular methods have been conducted in cattle (11, 15, 16). However, these reports were mainly carried out on cattle from the north of the country. In contrast, the epidemiology and risk factors associated, and the heterogeneity of *A. marginale* isolates remain largely unknown and understudied in the other geographic regions.

Therefore, the aims of this study were to evaluate the prevalence of *A. marginale* infection and their potential associated risk factors in cattle from 11 governorates belonging to eight bioclimatic areas from the north to the south of Tunisia and to characterize phylogeographically *A. marginale* isolates using two of the most phylogeographically informative markers (*lipA* and *sucB*). In addition, and considering the potential vaccinal interest of OmpA protein, we intend in our study to assess this protein conservation by comparing Tunisian sequences with those from other countries. In fact, various bioinformatics software were used in order to analyze the physicochemical properties, and the secondary and tertiary structures of their deduced proteins, and to predict their immunodominant epitopes of B and T cells.

## Materials and Methods

### Cattle and Site Description

**Figure 1:**
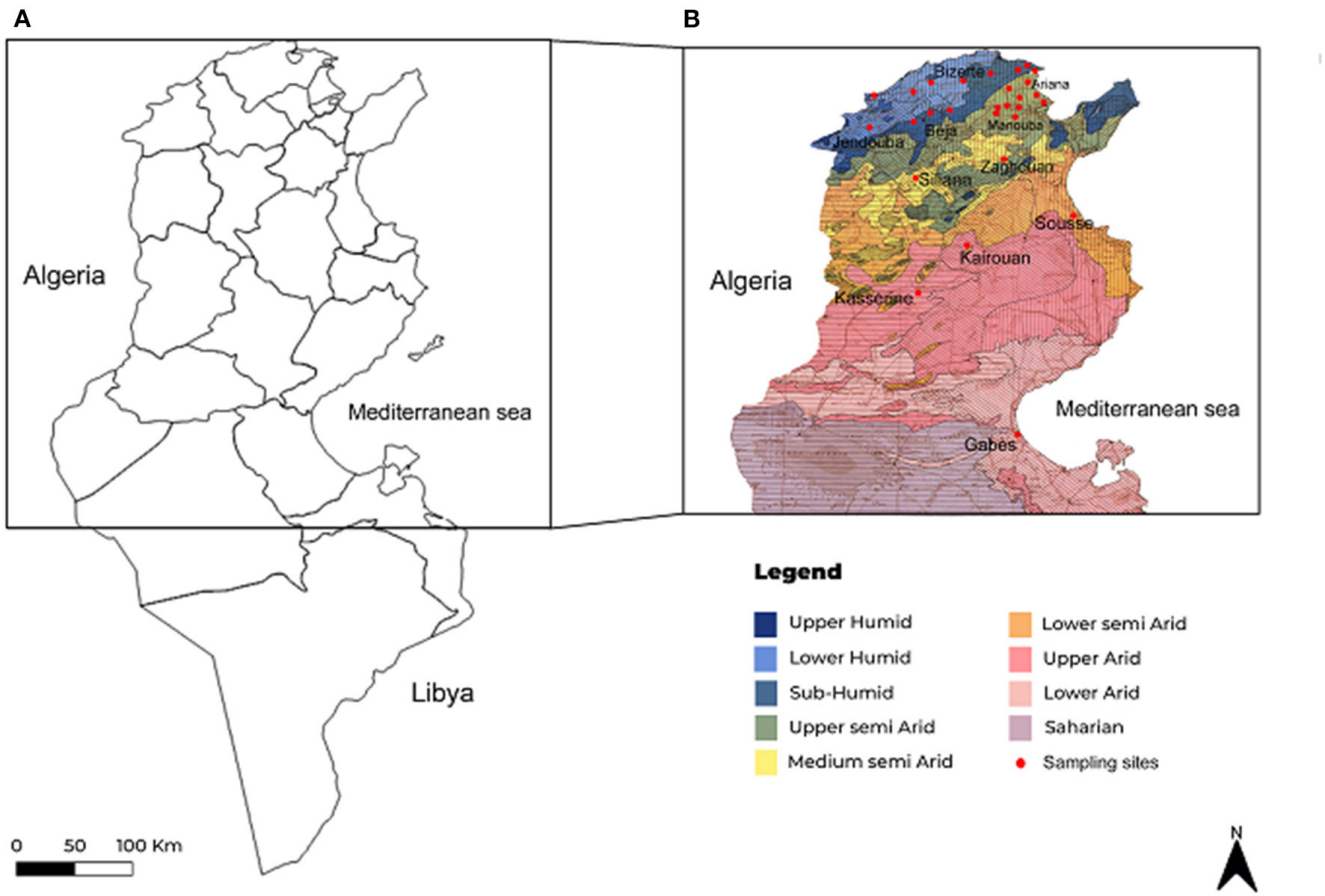
Maps showing geographical position of studied regions in Tunisia **(A)** and sampling sites presented in investigated regions according to bioclimatic stages **(B)**.

Between June and September 2019 and 2020, blood was randomly collected from 791 apparently healthy cattle (682 females and 109 males) reared in 165 farms located in 11 Tunisian governorates (Bizerte, Ariana, Manouba, Beja, Jendouba, Siliana, Kairouan, Kasserine, Zaghouan, Sousse, and Gabes) belonging to eight bioclimatic areas (subhumid, upper and lower humid, upper, middle, and lower semiarid, and upper and lower arid) (Figure 1 and Table 1). Visited farms are small, enclosing a mean of 20 bovine heads with traditional and poorly maintained housing facilities. Analyzed cattle were aged between 6 months and 15 years old, and the majority belonged to the Friesian Pie Noire and Holstein breeds. In spite of the use of acaricide treatment, 42.7% of the surveyed animals were infested with ticks, particularly in the mammary region and the inner surface of the ears (Table 1).

**Table 1:** Molecular prevalence rates of Anaplasmataceae and *Anaplasma marginale* according to geographic, bioclimatic, and cattle-related risk factors.

**Risk factors**	**Number**	**Anaplasmataceae**		***A. marginale***	
		**Positive (% ± CI^**a**^)**	***p-*Value**	**Positive (% ± CI^**a**^)**	***p-*Value**
**Governorates**					
Bizerte	189	53 (28.04 ± 0.064)	0.000^*^	12 (6.35 ± 0.034)	0.006^*^
Ariana	68	16 (23.53 ± 0.100)		1 (1.47 ± 0.027)	
Manouba	197	49 (24.87 ± 0.060)		1 (0.51 ± 0.009)	
Siliana	35	0 (0)		0 (0)	
Jendouba	62	11 (17.74 ± 0.095)		2 (3.23 ± 0.043)	
Beja	64	24 (37.50 ± 0.118)		2 (3.13 ± 0.042)	
Kairouan	30	4 (13.33 ± 0.121)		0 (0)	
Kasserine	24	2 (8.33 ± 0.110)		1 (4.17 ± 0.079)	
Zaghouan	42	6 (14.29 ± 0.105)		0 (0)	
Sousse	33	1 (3.03 ± 0.058)		0 (0)	
Gabes	47	0 (0)		0 (0)	
**Bioclimatic area**					
Subhumid	142	65 (45.77 ± 0.081)	0.000^*^	13 (9.15 ± 0.047)	0.000^*^
Upper humid	59	11 (18.64 ± 0.099)		2 (3.39 ± 0.045)	
Lower humid	35	8 (22.86 ± 0.138)		0	
Upper semiarid	344	69 (20.05 ± 0.042)		3 (0.87 ± 0.009)	
Medium semiarid	77	6 (7.79 ± 0.059)		0	
Lower semiarid	33	1 (3.03 ± 0.058)		0	
Upper arid	54	6 (11.11 ± 0.083)		1 (1.85 ± 0.035)	
Lower arid	47	0		0	
Total	791	166 (20.99 ± 0.028)		19 (2.40 ± 0.010)	
**Gender**					
Male	109	14 (12.84 ± 0.062)	0.024^*^	5 (4.59 ± 0.038)	0.108
Female	682	152 (22.29 ± 0.031)		14 (2.05 ± 0.010)	
**Age**					
≤ 2 years	212	33 (15.57 ± 0.048)	0.060	8 (3.77 ± 0.025)	0.214
>2 and ≤ 5 years	241	52 (21.58 ± 0.051)		3 (1.24 ± 0.013)	
>5 years	338	81 (23.96 ± 0.045)		8 (2.37 ± 0.015)	
**Breed**					
Friesian pie noire	287	34 (11.85 ± 0.037)	0.000^*^	7 (2.44 ± 0.017)	0.138
Holstein	257	74 (28.79 ± 0.055)		2 (0.78 ± 0.010)	
Brown Swiss	109	27 (24.77 ± 0.080)		4 (3.67 ± 0.034)	
Cross	95	20 (21.05 ± 0.081)		3 (3.16 ± 0.034)	
Local	20	5 (25 ± 0.189)		1 (5 ± 0.095)	
Other breeds^b^	23	6 (26.08 ± 0.180)		2 (8.69 ± 0.115)	
**Tick infestation**					
Infested	338	90 (26.63 ± 0.047)	0.000^*^	13 (3.85 ± 0.020)	0.022^*^
Not infested	453	76 (16.78 ± 0.034)		6 (1.32 ± 0.010)	
Total	791	166 (20.99 ± 0.028)		19 (2.40 ± 0.010)	

a *CI: 95% confidence interval*.

b *Other breeds are Tarentaise (n = 12), Montbeliarde (n = 9), Charolais (n = 1), and Belgian Blue White (n = 1)*.

* *Statistically significant test*.

### Blood Sampling and DNA Extraction

Whole blood samples were collected from the jugular vein of dairy cattle and placed into sterile tubes containing ethylenediaminetetraacetic acid (EDTA). For each animal, gender, age, and presence or absence of ticks were noted. DNA was extracted from 300 μl volume of EDTA-preserved whole blood using the Wizard® Genomic DNA purification kit (Promega, Madison, WI, USA) according to the manufacturer's instructions. DNA quality and quantity were evaluated with a Qubit® dsDNA assays (Thermo Fisher Scientific) and stored at −20°C until use.

### Molecular Detection of Anaplasmataceae and *Anaplasma marginale* Bacteria

EHR16SD and EHR16SR primers was used in a single PCR in order to detect all Anaplasmataceae bacteria by amplifying 345 bp of 16S rRNA gene with a thermal cycling profile as mentioned by Parola et al. (17) (Supplementary Table 1). Samples positive for Anaplasmataceae bacteria were used for the specific detection of *A. marginale* infection by using a single PCR with Amargmsp4F and Amargmsp4R primers amplifying 344 bp of *msp4* gene [(18); Supplementary Table 1]. For *A. marginale* genotyping and phylogeographic analysis, single PCRs were performed on *lipA* (538 bp) and *sucB* (808 bp) partial sequences used in MLST scheme developed earlier by Guillemi et al. (10), which showed better discriminative power than did other loci (14). The amplification profile was as described by Guillemi et al. (10). In order to assess the conservation of the OmpA protein vaccine candidate, the full *ompA* sequence (711 bp) was amplified with a single PCR by using AmOmpAF and AmOmpAR primers (Supplementary Table 1). The amplification conditions were as described by Futse et al. (8) (Supplementary Table 1). All PCRs were performed in a final volume of 50 μl containing 0.125 U/μl of Taq DNA polymerase (Biobasic Inc., Markham, ON, Canada), 1 × PCR buffer, 1.5 mM of MgCl_2_, 0.2 mM of dNTPs, 2 μl (50–150 ng) of genomic DNA, and 0.5 μM of primers. In this experiment, distilled water and DNA from bovine blood not infected with Anaplasmataceae bacteria, and DNA extracted from *A. marginale* (16) were used as negative and positive controls, respectively. PCR products were observed after electrophoretic migration in 1.5% agarose gels stained with ethidium bromide and under UV transillumination.

### Obtaining Sequences and Phylogenetic Analysis

By using the same primers as for the amplification, a selection of PCR products generated from *lipA, sucB*, and *ompA* partial sequences were sequenced in both directions after purification (Supplementary Table 1). The reaction was carried out by using a conventional Big Dye Terminator cycle sequencing ready reaction kit (Perkin Elmer, Applied Biosystems, Foster City, CA, USA) and an ABI3730XL automated DNA sequencer. Chromatograms were edited with Chromas Lite v 2.01. The DNAMAN program (Version 5.2.2; Lynnon Biosoft, Quebec, QC, Canada) was used to perform multiple sequence alignment and to translate nucleotide to amino acid sequences. BLAST analysis (http://blast.ncbi.nlm.nih.gov) was carried out to search nucleotide similarity (19). DNAMAN program was also used to estimate genetic distances calculated by using the maximum composite likelihood method (20). Phylogenetic trees were built by neighbor-joining method integrated in the same software (21). Robustness rates of the internal branches' nodes were calculated based on a statistical support of 1,000 reiterations. Indeed, a total of 64, 76, and 42 *A. marginale* partial sequences, respectively, of *lipA, sucB*, and *ompA* genes from GenBank were included in this analysis. Obtained partial sequences from *A. marginale* Tunisian isolates were deposited under GenBank under accession numbers MZ221566 to MZ221579, MZ221580 to MZ221586, and MZ221587 to MZ221597 for *lipA, sucB*, and *ompA* genes, respectively.

### Statistical Analysis

Exact confidence intervals (CIs) for prevalence rates at the 95% level were calculated. χ^2^ and Fisher's exact tests integrated in the Epi Info 6.01 software (CDC, Atlanta) were used to perform a comparison of Anaplasmataceae and *A. marginale* prevalence rates among different categories for each risk factor and among different governorates and bioclimatic areas. Observed differences were considered to be statistically significant at a 0.05 threshold value. A chi square Mantel–Haenszel test was carried out in order to take into consideration any confusion factor.

### OmpA Protein Conservation Assessment

#### Analysis of OmpA Protein Properties

ProtParam, a freely accessed online server (http://web.expasy.org/protparam/), was used to determine protein properties such as molecular weight, amino acid composition, isoelectric point (pI), stability index, grand average of hydropathicity (GRAVY), and estimated half-life. By using default parameters, the secondary structure of the protein was predicted from primary protein sequence by SOPMA online server (https://npsa-prabi.ibcp.fr/cgi-bin/npsa_automat.pl?page=/NPSA/npsa_sopma.html).

#### Prediction of N-Glycosylation Sites, Transmembrane Topology, and Disulfide Bonds

*N*-Glycosylation sites in protein were predicted using NetNGlyc1.0 server (http://www.cbs.dtu.dk/services/NetNGlyc/). Transmembrane helices were predicted using TMHMM server 2.0 [http://www.cbs.dtu.dk/services/TMHMM/; (22)]. Disulfide bonds in protein were determined using DiANNA 1.1 web server [http://clavius.bc.edu/~clotelab/DiANNA/; (23)].

#### Antigenicity and Allergenicity Prediction

Antigenicity of OmpA protein was predicted by using VaxiJen 2.0 server (http://www.ddg-pharmfac.net/vaxijen/VaxiJen/VaxiJen.html) with default threshold of 0.5 for the determination of the antigenic protein (24). This server is used in order to predict the most antigenic proteins serving to be protective antigens or subunit vaccines.

AllerCatPro, a freely accessed online server, was used to determine the allergenicity of our proposed protein for vaccine development (25). This server predicts the allergenic potential of proteins based on similarity of their 3D protein structure and their amino acid sequence compared with those of known protein allergens (25).

#### B-Cell Epitope Identification

The search for potentially immunogenic epitopes in the analyzed protein sequence is generally carried out in order to identify epitopes essential for the creation of an effective vaccine. This approach considerably reduces the experiments leading to the design of vaccines and to the creation of an immunodiagnostic method. The aim of this prediction is to select the epitopes in a given antigen that would interact with B lymphocytes and initiate an immune response (26).

Linear B-cell epitopes were predicted using ABCpred [http://www.imtech.res.in/raghava/abcpred/; (27)] and Bepipred [http://tools.immuneepitope.org/bcell/; (28)] servers, and the epitopes in common were selected. For determining conformational epitopes, the OmpA protein sequences were submitted to the CBTope [http://www.imtech.res.in/raghava/cbtope/; (29)] and BepiPred 2.0 (30); web servers and the common epitopes that predict using these two programs were considered. The default settings were applied to all the tools used.

The predicted linear and conformational B-cell epitopes were further analyzed using the following tools: Emini et al. surface accessibility prediction (31), Fasman and Chou beta-turn prediction (32), Karplus and Schulz flexibility prediction (33), Parker et al. hydrophilicity prediction (34), and Kolaskar and Tongaonkar antigenicity (35). All these tools are available at IEDB analysis resource (http://tools.immuneepitope.org/bcell/), and the default settings were applied to all the used tools.

#### T-Cell Epitope Identification

The identification of the major histocompatibility complex class I (MHC class I) T-cell epitopes was performed by using the NetCTL 1.2 server (36). The selection method is based on the peptide MHC I binding, proteasomal C terminal cleavage, and transporter associated with antigen processing (TAP) transport efficiency. Epitope prediction was limited to 12 MHC-I supertypes. MHC-I binding and proteasomal cleavage were obtained via artificial neural networks, and the weight matrix was employed for the efficiency of TAP transport. The parameter used for this analysis was set at the threshold of 0.5 in order to obtain a sensitivity and specificity of 0.89 and 0.94, respectively, allowing the prediction of more epitopes for further analysis. A combined algorithm of MHC-I binding, TAP transport efficiency, and proteasomal cleavage efficiency was used to predict overall scores (36).

By using the IEDB server (http://tools.immuneepitope.org/mhcii/), MHC class II T-cell epitopes were predicted. The selection IEDB Recommended uses the Consensus approach (37), combining NN-align (38, 39), SMM-align (40), and CombLib and Sturniolo et al. (41) if any corresponding predictor is available for the molecule; otherwise, NetMHCIIpan is used (39, 42). The Consensus approach considers a combination of any three of the four methods, if available, where Sturniolo is a final choice. The predictive performances are based on large-scale evaluations of the performance of the MHC class II binding predictions (37, 43, 44).

The antigenicity of the predicted T-cell epitopes was assessed using the Kolaskar and Tongaonkar antigenicity (35) belonging to the IEDB analysis resource (http://tools.immuneepitope.org/bcell/) using the standard threshold value of 1.030.

#### Epitope Conservancy Analysis

The conservation of predicted epitopes among different isolates was analyzed using IEDB epitope conservancy analysis tool [http://tools.immuneepitope.org/tools/conservancy/iedb input; (45)].

#### Three-Dimensional Structure Prediction, Refinement, and Validation of OmpA Protein

The three-dimensional structure prediction of OmpA protein was performed by using I-TASSER at http://zhanglab.ccmb.med.umich.edu/I-TASSER/ (46). I-TASSER uses a hierarchical approach to predict protein structure and function. A confidence score (C-score) in the range of (−5, 2) was used to evaluate the quality of the modeled structures, and a high C-score confirms a high confidence model. Pymol 1.7 was used to visualize the tertiary structures after modeling (47). After selecting the best three-dimensional models according to the C-score, the ModRefiner server (http://zhanglab.ccmb.med.umich.edu/ModRefiner/) was used to refine the selected structures (48). The quality of the refined structures was assessed by Ramachandran plot, by using the PROCHECK function (49) in PDBSUM tool (https://www.ebi.ac.uk/thorntonsrv/databases/pdbsum/Generate.html). The backbone conformation of the protein structures was verified by analyzing φ (Phi) and ψ (Psi) dihedral angles for each residue and finally classifies the residues into favorable, allowed, and outlier regions well-visualized in the Ramachandran plot (50). The selected B and T epitopes were schematically showed in their protein region after three-dimensional prediction using Pymol 1.7 (47).

## Results

### Molecular Prevalences of Anaplasmataceae and *Anaplasma marginale*

The overall prevalence rates of Anaplasmataceae and *A. marginale* were 20.99 and 2.40%, respectively. The statistically highest molecular prevalence of Anaplasmataceae is observed in cattle belonging to the governorate of Beja (37.50%, 24/64), while those belonging to the governorate of Sousse are the least infected (3.03%, 1/33) (*p* < 0.001). In addition, no cattle belonging to Siliana and Gabes governorates were infected with Anaplasmataceae bacteria. The highest molecular prevalence of *A. marginale* is recorded in cattle belonging to the governorate of Bizerte (6.35%, 28/189), while those from Manouba are the least infected (0.51%, 1/197). In addition, cattle belonging to the governorates of Siliana, Kairouan, Zaghouan, Sousse, and Gabes were not infected with this bacterial species (*p* = 0.006; Table 1).

The statistically highest molecular prevalence rates of Anaplasmataceae and *A. marginale* are recorded in cattle from the subhumid bioclimatic area estimated, respectively, at 45.77% (65/142) and 9.15% (13/142) (*p* < 0.001). Furthermore, females (22.29%, 152/682) are more infected with Anaplasmataceae than males (12.84%, 14/109) (*p* = 0.022). Holstein cattle (28.79%, 74/257) were significantly more infected with Anaplasmataceae (*p* < 0.001) than other breeds (Table 1). Cattle infested by ticks were statistically more infected with Anaplasmataceae (26.63%, 90/338) and *A. marginale* (3.85%, 13/338) than were those free of ticks [16.78% (76/453) and 1.32% (6/453), respectively] (*p* < 0.001 and *p* = 0.022, respectively; Table 1).

### Genotyping and Diversity Analysis

#### *Anaplasma marginale* LipA Partial Sequences

**Figure 2:**
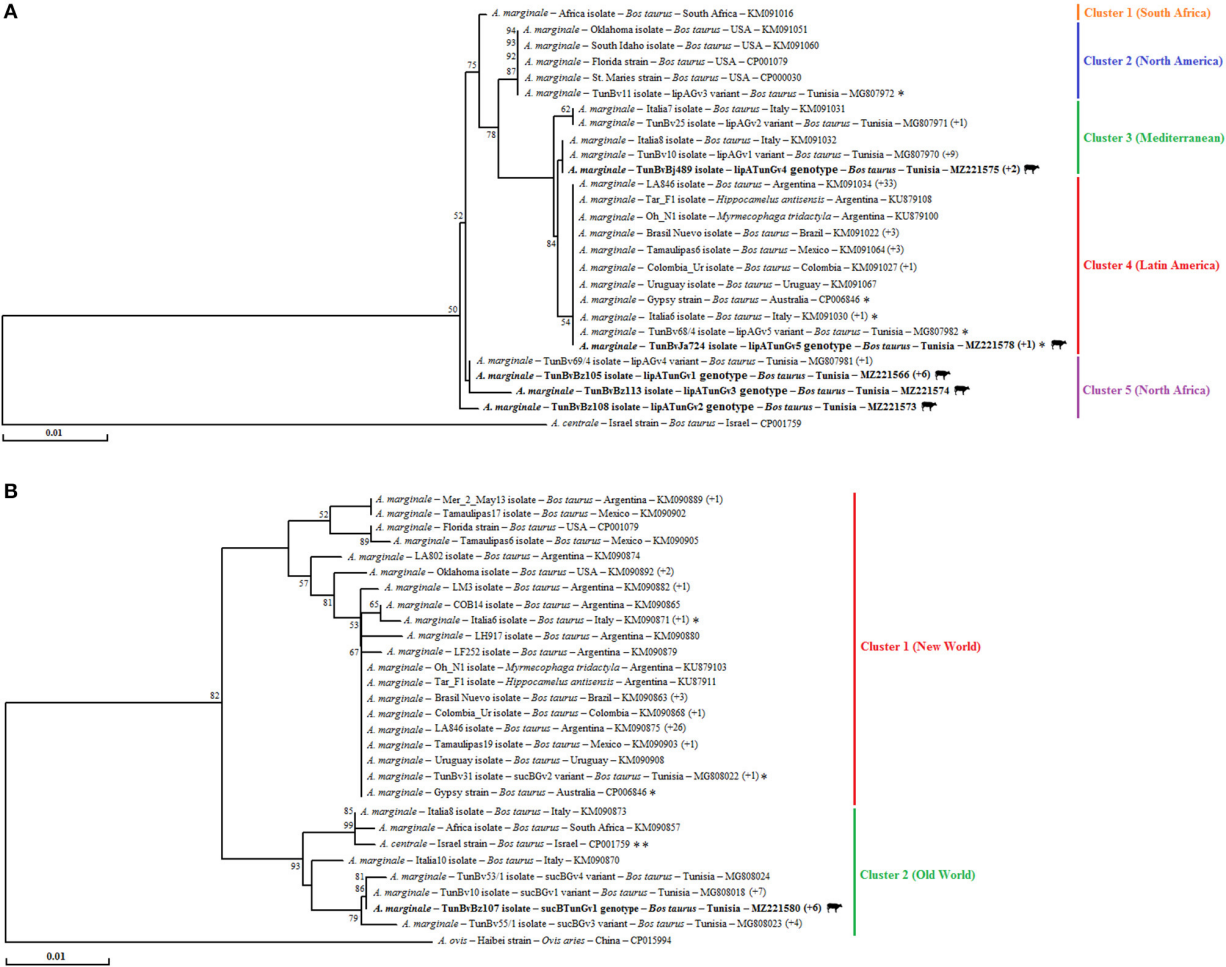
Phylogenetic tree showing all *Anaplasma marginale* genetic variants based on multiple alignment of *lipA* **(A)** and *sucB* **(B)** partial nucleotide sequences (501 and 681 bp, respectively) using the “neighbor-joining” method. The numbers related to the nodes represent robustness rates over 1,000 iterations supporting the nodes (only rates >50% are shown). The host, strain or isolate, country of origin, and GenBank accession number are indicated. The sequences of *A. marginale* newly obtained in the present study are in bold and marked with a bovine picture. Sequences that are not classified into their appropriate geographic regions represented by clusters are indicated with an asterisk. The numbers that are in parentheses at the end of some sequences represent isolates or strains that are represented by an identical sequence from the isolate or strain present in the tree.

A total of five distinct genotypes (lipATunGv1 to lipATunGv5), which differ in nine nucleotide positions, were identified after the alignment of *lipA* partial nucleotidic sequences (501 bp) of 14 Tunisian isolates (Table 2 and Supplementary Tables 2, 3). Two genotypes (lipATunGv2 and lipATunGv3) showed nucleotide diversity as compared with all sequences presented in GenBank and are recognized to be new genetic variants (Figure 2A and Table 2). Nucleotide sequence homology rates between *lipA* genotypes obtained in this study were 98.4–99.8% (100% at the protein level) (Supplementary Table 2). Additionally, Tunisian genotypes were 98.4–100% similar to all *A. marginale* sequences analyzed in the phylogenetic tree (Figure 2A and Supplementary Table 2). Nucleotide homology rates decreased (86.2–87.2%) when the Tunisian variants are compared with the *Anaplasma centrale* reference sequence (CP001759) published in GenBank.

**Table 2:** Designation and information about sequencing of *Anaplasma marginale* genotypes identified in this study.

**Gene**	**Isolate**	**Governorate (district)**	**GenBank^**a**^**	**Genotype**	**BLAST analysis**
*lipA*	TunBvBz105	Bizerte (Utique)	MZ221566	lipATunGv1	100% MG807984
	TunBvBz106	Bizerte (Utique)	MZ221567		
	TunBvBz107	Bizerte (Utique)	MZ221568		
	TunBvAr312	Ariana (Raoued)	MZ221569		
	TunBvBz346	Bizerte (El Alia)	MZ221570		
	TunBvBz271	Bizerte (Ras Jebel)	MZ221571		
	TunBvBz362	Bizerte (Ghar El Melh)	MZ221572		
	TunBvBz108	Bizerte (Utique)	MZ221573	lipATunGv2	99.8% MG807984
	TunBvBz113	Bizerte (Utique)	MZ221574	lipATunGv3	99.4% MG807984
	TunBvBj489	Beja (Amdoun)	MZ221575	lipATunGv4	100% MG807970
	TunBvBj488	Beja (Amdoun)	MZ221576		
	TunBvBz274	Bizerte (Ras Jebel)	MZ221577		
	TunBvJa724	Jendouba (Tabarka)	MZ221578	lipATunGv5	100% MG807982
	TunBvJa742	Jendouba (Tabarka)	MZ221579		
*sucB*	TunBvBz107	Bizerte (Utique)	MZ221580	sucBTunGv1	100% MG808018
	TunBvAr312	Ariana (Raoued)	MZ221581		
	TunBvBz346	Bizerte (El Alia)	MZ221582		
	TunBvBz362	Bizerte (Ghar El Melh)	MZ221583		
	TunBvJa724	Jendouba (Tabarka)	MZ221584		
	TunBvJa742	Jendouba (Tabarka)	MZ221585		
	TunBvBz105	Bizerte (Utique)	MZ221586		
*ompA*	TunBvBz105	Bizerte (Utique)	MZ221587	ompATunGv1	100% CP000030
	TunBvBz107	Bizerte (Utique)	MZ221588		
	TunBvBz108	Bizerte (Utique)	MZ221589		
	TunBvBz113	Bizerte (Utique)	MZ221590		
	TunBvBj488	Beja (Amdoun)	MZ221591		
	TunBvBz346	Bizerte (El Alia)	MZ221592		
	TunBvAr312	Ariana (Hessiene)	MZ221593		
	TunBvBz362	Bizerte (Aousseja)	MZ221594		
	TunBvBz361	Bizerte (Aousseja)	MZ221595		
	TunBvKa639	Kasserine (Sebitla)	MZ221596		
	TunBvBz106	Bizerte (Utique)	MZ221597	ompATunGv2	99.9% CP000030

*MG807984 is the GenBank accession number of the isolate “TunBv74/1” infecting cattle from Tunisia, and CP000030 is the GenBank accession number of the “St. Maries” strain infecting cattle in the United States*.

a *GenBank accession number*.

Multiple alignments of the five Tunisian genotypes, representing the 14 sequenced isolates, with the 73 *A. marginale* partial sequences obtained from GenBank, generated a phylogenetic tree made up of five main clusters (Figure 2A). The first cluster is formed exclusively by the South African strain. The second cluster consists mainly of isolates from North America (represented exclusively by the United States). The third cluster is formed by Mediterranean isolates from Italy and Tunisia. The fourth cluster mainly contains strains from Latin America like Mexico, Brazil, Colombia, Uruguay, and Argentina. The last cluster includes isolates infecting cattle located in North Africa represented until this study by Tunisia. The last cluster includes isolates exclusively infecting Tunisian cattle (Figure 2A).

Our isolates were assigned to the last three clusters (one genotype in the third and fourth clusters and three in the fifth). Particularly, the genotypes lipATunGv1 to lipATunGv3 grouped together in the fifth cluster with other isolates reported earlier in Tunisia. Within the third cluster, the lipATunGv4 genotype is included with other Mediterranean isolates from Tunisia and Italy. In the fourth cluster, the lipATunGv5 genotype was identical to numerous isolates mainly originating from Latin America such as Argentina, Brazil, and Mexico (Figure 2A).

#### *Anaplasma marginale SucB* Partial Sequences

Seven *sucB* partial sequences (681 bp) of the *A. marginale* Tunisian isolates were aligned, allowing the identification of a single genotype (sucBTunGv1) (Table 2). The sucBTunGv1 genotype was 97.5–100% identical to all *A. marginale* sequences analyzed in the phylogenetic tree giving homology rates between 96.9 and 100% at the protein level (Supplementary Table 4). In fact, 27 single-nucleotide polymorphisms (SNPs) were observed giving eight amino acid substitutions (Supplementary Figure 1). By comparing the revealed genotype to *A. centrale* reference sequence (CP001759), the identity rate was 98.7%.

The phylogenetic tree based on the alignment of our genotype with all *sucB* partial sequences of *A. marginale* found in GenBank and one *A. centrale* reference sequence added as an outgroup shows the occurrence of two main clusters (Figure 2B). Except the two Italian isolates (Italia6 and Italia7), the Tunisian TunBv31 isolate and the Australian strain (Gypsy), the first cluster includes all isolates from New World countries (i.e., United States, Mexico, Brazil, Colombia, Uruguay, and Argentina). The second cluster contains several isolates from Old World countries like Italy (isolates Italia8 and Italia10) and South Africa (isolate Africa) in addition to the Israeli *A. centrale* strain (Figure 2B). The sucBTunGv1 genotype representing the seven Tunisian revealed that isolates were assigned to the second cluster (Figure 2B). Within this cluster, sucBTunGv1 forms, with other previously revealed Tunisian isolates, a distinct subcluster relatively distant from the *A. centrale* reference strain grouped with other isolates from the Old World countries only represented by Italy (isolates Italia8 and Italia10) and South Africa (isolate Africa) (Figure 2B).

#### *Anaplasma marginale OmpA* Partial Sequences

Partial *ompA* sequences (679 bp) of the 11 Tunisian *A. marginale* isolates revealed in this study were aligned, allowing the identification of two different genotypes (ompATunGv1 and ompATunGv2) (Table 2). The ompATunGv1 genotype was found to be identical to the “St. Maries” strain (GenBank accession number CP000030) infecting cattle in United States, and the ompATunGv2 genotype showed a degree of nucleotide diversity compared with all *ompA* sequences published in GenBank and was considered as a novel genetic variant (Table 2). Nucleotide homology rates between the two genotypes obtained in this study and all the other available genetic variants were from 99.1 to 99.9% (98.2–100% at the amino acid sequence) (Supplementary Table 5). In fact, nine SNPs were observed representing five non-synonymous substitutions (Supplementary Table 6). By comparing our genotypes ompATunGv1 and ompATunGv2 with those belonging to the species closest to *A. marginale*, sequence identity was more important with *Anaplasma ovis* reference sequence (CP015994) (85.0 and 85.2%) compared with that of *A. centrale* (CP001759) (81.5 and 81.3%, respectively).

**Figure 3:**
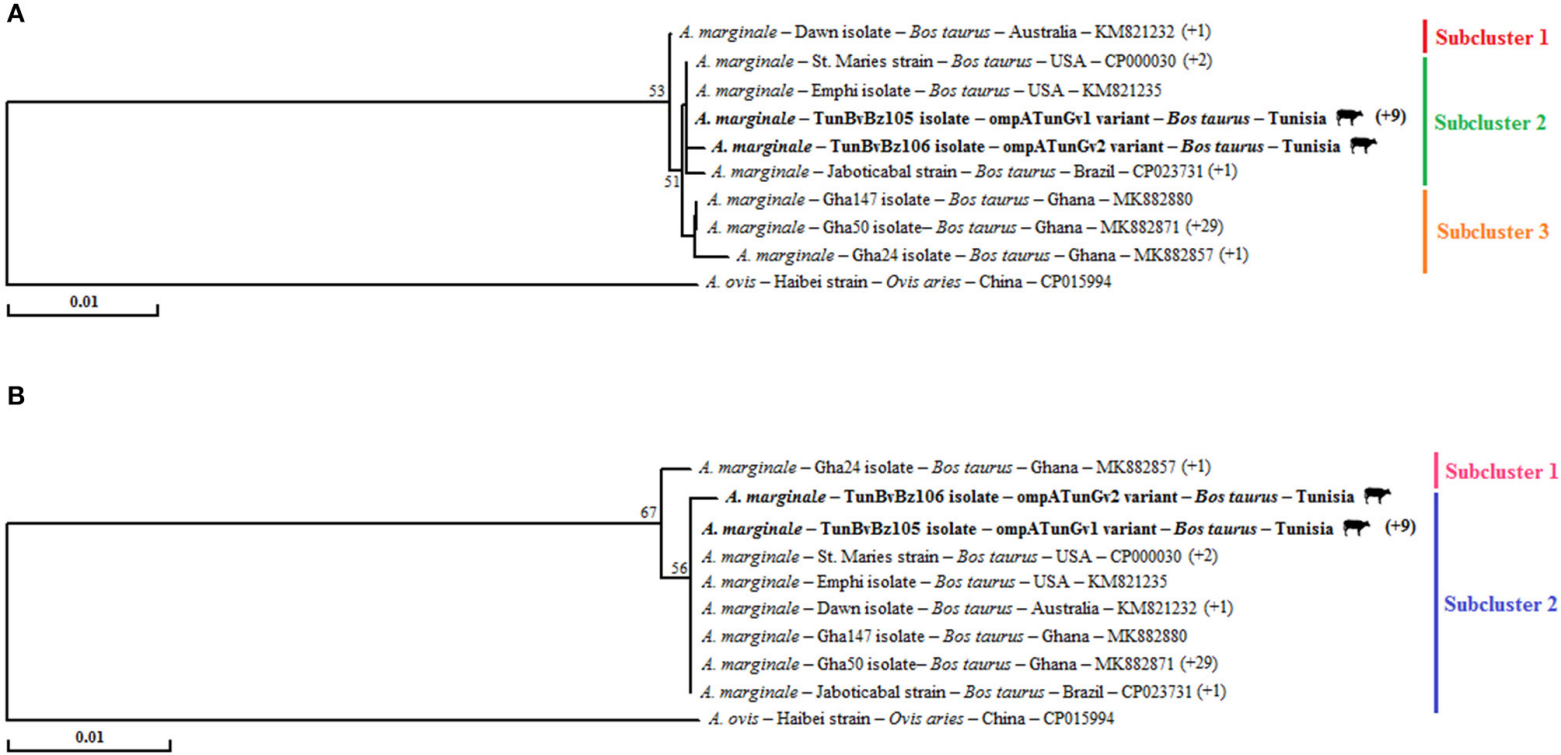
Phylogenetic analysis of all available genetic variants of *Anaplasma marginale* based on the multiple alignment of partial nucleotide sequences (679 bp) of *ompA* gene **(A)** and their deduced amino acid sequences (236 AA) of the OmpA protein **(B)** using the “neighbor-joining” method. The numbers related to the nodes represent the robustness rates over 1,000 iterations (only the rates >50% are represented). The host, strain or isolate, country of origin, and GenBank accession number are listed. The sequences of *A. marginale* newly obtained in this study are in bold and marked with a bovine picture. The numbers that are in parentheses at the end of some sequences represent isolates or strains that are represented by an identical sequence from the isolate or strain present in the tree.

For this gene, phylogenetic trees based on the alignment nucleotide and amino acid sequences of our *A. marginale* isolates with those found in GenBank show the presence of a single cluster (Figures 3A,B). This finding was confirmed by calculating the rates of concerned nodes, which did not exceed 53 and 67%, respectively, based on nucleotide and amino acid sequences (Figures 3A,B). In tree based on nucleotide sequences, this unique cluster is formed by three subclusters. The first includes one isolate and one strain from Australia. The second is formed with all isolates and strains from New World countries like the United States and Brazil. The third subcluster contains all the Ghanaian isolates (Figure 3A). Tunisian isolates were assigned to the second subcluster. In particular, the ompATunGv1 genotype is grouped with all strains originating from the United States, while the ompATunGv2 genotype is classified separately in this same subcluster (Figure 3A). In the tree based on amino acid sequences, the unique cluster is composed by two subclusters. The first formed with only one Ghanaian isolate (Gha24, MK882857). The second is formed with all isolates from Tunisia, isolates and strains from New World countries like the United States and Brazil, and the remaining Ghanaian isolates (Figure 3B).

### OmpA Protein Conservation Assessment

#### OmpA Protein Properties

By taking the amino acid sequence deduced from the genetic variant V1 (MK882880) as a reference, the primary OmpA amino acid sequence was given as input for analysis by using ExPASy ProtParam tool. OmpA protein consisted of 236 amino acids with 13.1% of positively charged amino acids (arginine and lysine) and 16.1% of negatively charged amino acids (aspartate and glutamate). The molecular weight of the protein was estimated to be about 25,686 Da. The theoretical isoelectric point (pI) was about 5.32, predicting that protein is negatively charged at neutral pH. The extinction coefficient of the protein was estimated considering water as solvent at 280 nm. The extinction coefficients were computed to be 17,210 and 16,960 M^−1^ cm^−1^ when assuming all pairs of cysteines form cystines and when considering all pairs of cysteines are reduced, respectively.

The estimated half-lives of the protein were 30, >20, and >10 h in mammalian reticulocytes (*in vitro*), yeast (*in vivo*), and *Escherichia coli* (*in vivo*), respectively. The instability index was predicted to be 55.55, classifying it to be an unstable protein. The aliphatic index was estimated to be 79.75, and GRAVY was calculated to be −0.317.

**Figure 4:**
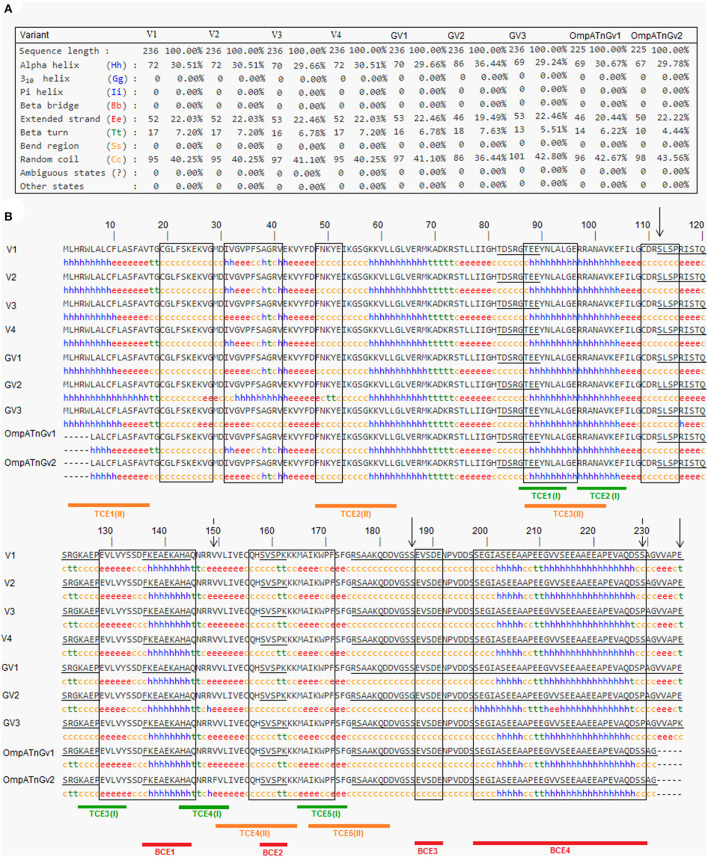
Information on the secondary structure composition of OmpA proteins deduced from the nine genetic variants representing all *Anaplasma marginale* strains and isolates available in GenBank **(A)** and multiple alignment of predicted amino acid sequences and secondary structures of the OmpA proteins representing all *A. marginale* strains and isolates available in GenBank **(B)**. Sequential and conformational epitopes linked to B cells are, respectively, underlined and boxed. Red lines represent epitopes (BCE1–BCE4), which are in common between sequential and conformational epitopes. The green and orange lines represent the epitopes [TCE1(I)–TCE1(I)] linked to MHC class I T cells and the epitopes [TCE1(II)–TCE1(II)] binding to MHC class II T cells. The positions where there are amino acid substitutions between the analyzed protein sequences are represented by descending arrows.

SOPMA secondary structure prediction method was used to analyze the secondary structure of OmpA protein by giving the primary sequence of the protein as input. Other parameters were studied by setting the default threshold values. The protein was predicted to be made of 30.51% of alpha-helix (Hh), 22.03% of extended strand (Ee), 7.20% of beta-turn (Tt), and 40.25% of random coil (Cc) (Figure 4).

#### Predicted Disulfide Bonds in OmpA Protein

DIANNA 1.0 web server was employed to predict the disulfide bonds in protein. This server predicted two disulfide bonds by giving primary amino acid sequence as input. The server algorithm consists of five steps. In the first step, the submitted input sequence was executed in PSIBLAST. In step 2, the secondary structure of the protein was predicted using PSIPRED. In step 3, the oxidation state of the disulfide was estimated. In step 4, the disulfide bonds were computed using a diresidue neural network. In the end, the predicted disulfide bonds were weighted by Ed Rothberg's implementation of the Edmonds–Gabow maximum weight matching algorithm. Disulfide bonds were to be formed between positions 9–155 and 19–109.

#### Transmembrane Helices and N-Glycosylation Sites in OmpA Protein

Transmembrane helices were predicted by using the TMHMM server. Indeed, an absence of transmembrane helices was noted in OmpA protein. Asparagine in NXS/T sequence, where N is asparagine, X is any amino acid, S is serine, and T is threonine, is generally glycosylated in proteins produced by eukaryotes, archaea, and very rarely bacteria. In addition, a prediction of *N*-glycosylation sites in OmpA protein was performed by the NetNGlyc 1.0 server. A default potential threshold of 0.5 was considered for this analysis, which showed a total absence of glycosylation sites in all OmpA proteins deduced from all available genetic variants.

#### Antigenicity and Allergenicity of OmpA Whole Protein

Estimation of antigenicity and allergenicity are crucial in the selection of the candidate protein, which can be used in a vaccine using unilamellar liposomes. The antigenicity of the OmpA protein was predicted by using the VaxiJen v2.0 server by adding the primary sequence of the protein as an additional input at a threshold of 0.5 and with the selection of bacteria as model. The server predicted that OmpA protein can be an antigen with an overall antigenicity prediction score of 0.7675.

Allergenicity of OmpA protein was obtained from AllerCatPro online server by giving the primary sequence of the protein as an entry. A weak evidence of allergenicity for this protein was predicted with a rate of 100%.

#### Tertiary Structure Prediction, Refinement, and Validation of the OmpA Protein

Five 3-dimensional structures were predicted for the OmpA protein sequence by using the I-TASSER server. Of these, the best structure was selected according to the highest C-score. The resulting structure was refined by using the ModRefiner server. The best refined model was selected based on the results of the Ramachandran plot (Supplementary Figure 2B). In fact, 48.5, 36.4, 11.1, and 4.0% of the residuals in the initial model were, respectively, in main, authorized, generous, and non-authorized regions. However, in the best refined model, 74.2, 21.7, 2, and 2% of the tailings were, respectively, in the main, authorized, generous, and prohibited regions (Supplementary Figure 2B).

#### Predicted B-Cell Epitopes of OmpA Protein

Based on ABCpred and BepiPred servers, eight common linear epitopes (SE1–SE8) are selected (Table 3). In addition, nine conformational B-cell epitopes (CE1–CE8) of OmpA protein were predicted using Bepipred 2.0 and CBTope programs (Table 4). By combining the results from the four servers, four epitopes (BCE1–BCE4) common between B-cell linear and conformational peptides were obtained from *A. marginale* OmpA protein (Table 5). By analyzing the secondary structures of the proteins deduced from the nine genetic variants, all the sequential and/or conformational epitopes selected except SE3, SE7, and CE4 contain high proportions of extended strands and random coils (Figure 4).

**Table 3:** Linear epitopes of the OmpA protein of *Anaplasma marginale* predicted using BepiPred and ABCpred and epitope conservancy result.

**Number**	**Name**	**Start**	**End**	**Epitope sequences**	**Length**	**Epitope conservancy (conserved sequence/total)**
1	SE1	82	89	TDSRGTEE	8	100% (9/9)
2	SE2	113	128	S(L)LSPRISTQSRGKAEP	16	88.9% (8/9)
3	SE3	136	144	FKEAEKAHA	9	100% (9/9)
4	SE4	158	162	SVSPK	5	100% (9/9)
5	SE5	175	192	RSAAKQDDVGSS(G)EVSDEN	18	88.9% (8/9)
6	SE6	193	210	PVDDSSEGIASEEAAPEE	18	100% (9/9)
7	SE7	211	228	GVVSEEAAEEAPEVAQDS	18	100% (9/9)
8	SE8	229	236	S(P)AGVVAPE(K)	8	55.6% (5/9)

*The reference sequence considered in this analysis is that deduced from the genetic variant V1 (GenBank accession number MK882880). The letters found in parentheses represent the amino acids substituted in at least one of the proteins deduced from genetic variants other than the reference variant V1*.

**Table 4:** B-cell conformational epitopes of the OmpA protein of *Anaplasma marginale* predicted using BepiPred 2.0 and CBTope and epitope conservancy result.

**Number**	**Name**	**Start**	**End**	**Epitope sequences**	**Length**	**Epitope conservancy (conserved sequence/total)**
1	CE1	19	28	CGLFSKEKVG	10	100% (9/9)
2	CE2	31	41	IVGVPFSAGRV	11	100% (9/9)
3	CE3	48	52	FNKYE	5	100% (9/9)
4	CE4	87	96	TEEYNLALGE	10	100% (9/9)
5	CE5	109	115	CDRS(L)LSP	7	88.9% (8/9)
6	CE6	128	145	EVLVYSSDFKEAEKAHAQ	18	100% (9/9)
7	CE7	156	171	QHSVSPKKKMAIKWPF	16	100% (9/9)
8	CE8	187	191	EVSDE	5	100% (9/9)
9	CE9	198	229	SEGIASEEAAPEEGVVSEEAAEEAPEVAQDSS(P)	32	55.6% (5/9)

*The reference sequence considered in this analysis is that deduced from the genetic variant V1 (GenBank accession number MK882880). The letters found in parentheses represent the amino acids substituted in at least one of the proteins deduced from genetic variants other than the reference variant V1*.

**Table 5:** Common epitopes between B-cell linear and conformational peptides obtained from *Anaplasma marginale* OmpA protein by using BepiPred, ABCpred, BepiPred 2.0 and CBTope, and epitope conservancy result.

**Number**	**Name**	**Start**	**End**	**Epitope sequences**	**Length**	**Epitope conservancy (conserved sequence/total)**
1	BCE1	136	144	FKEAEKAHA	9	100% (9/9)
2	BCE2	158	162	SVSPK	5	100% (9/9)
8	BCE3	187	191	EVSDE	5	100% (9/9)
9	BCE4	198	229	SEGIASEEAAPEEGVVSEEAAEEAPEVAQDSS(P)	32	55.6% (5/9)

*The reference sequence considered in this analysis is that deduced from the genetic variant V1 (GenBank accession number MK882880). The letters found in parentheses represent the amino acids substituted in at least one of the proteins deduced from genetic variants other than the reference variant V1*.

All of these epitopes were analyzed for a set of factors that determine the potentiality for B-cell epitopes (e.g., surface accessibility, hydrophilicity, secondary structures, flexibility, and antigenicity). Combining the results of hydrophilicity (Supplementary Figure 3A), beta-turn (Supplementary Figure 3B), surface accessibility (Supplementary Figure 3C), and flexibility (Supplementary Figure 3D), we found that all revealed sequential and/or conformational epitopes could be potential B-cell epitopes. By analyzing the antigenicity results from Kolaskar and Tongaonkar, we found that all epitopes are potentially antigenic except for the CE3 and BCE1 epitopes (Supplementary Figure 4).

**Figure 5:**
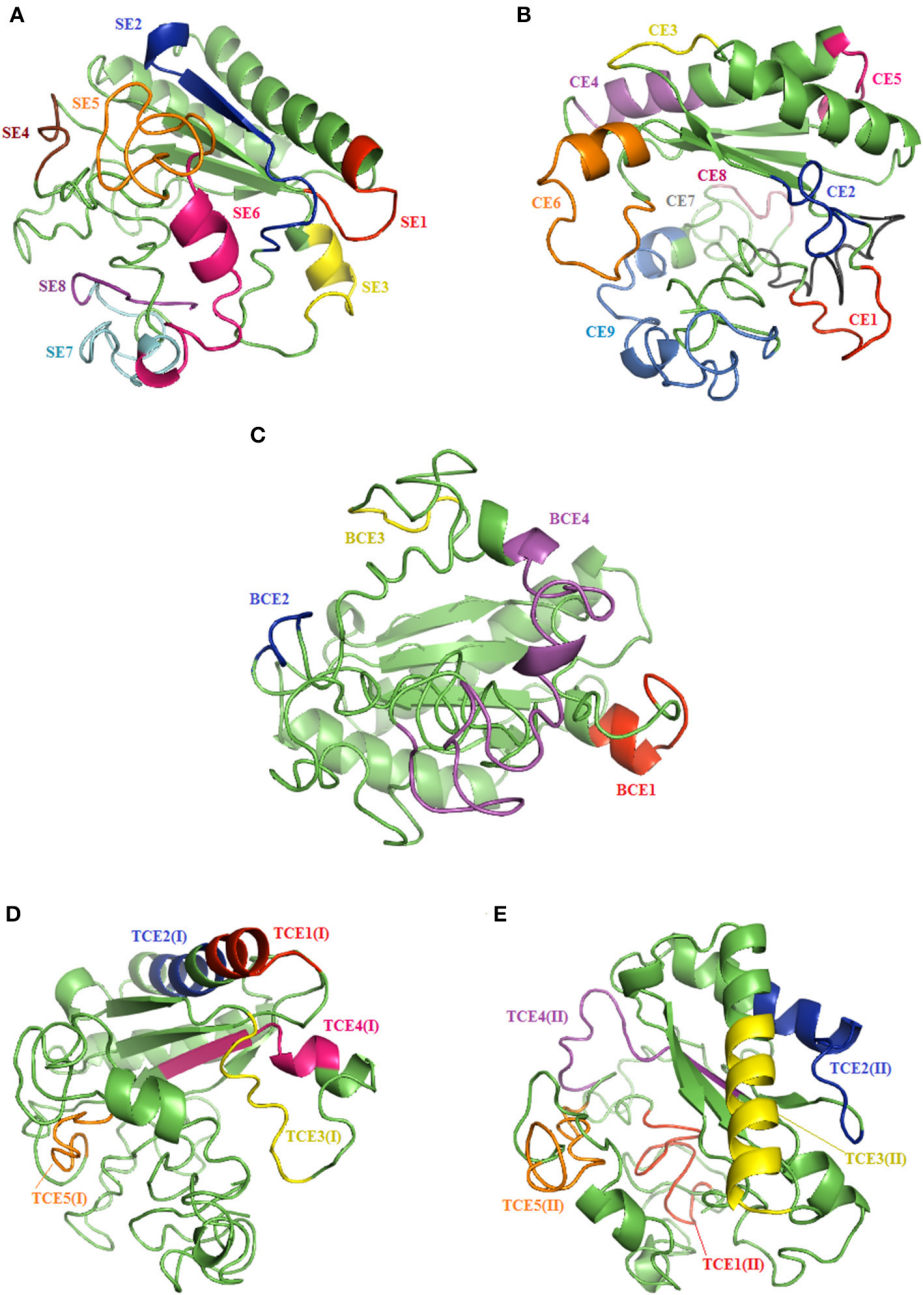
Results of the tertiary structure prediction of the reference OmpA protein (V1) showing linear **(A)**, conformational **(B)**, and common **(C)** epitopes bound to B cells, and MHC class I **(D)** and II **(E)** T-cell epitopes.

The IEDB conservancy analysis tool analyzed the conservancy of the predicted B-cell epitopes, which are presented in Tables 3–5. The results showed that the epitope conservation rate was 100% for most epitopes, allowing their total preservation within the different *A. marginale* isolates, while, for the rest of the epitopes for which the conservation rates are 88.9 and 55.6%, the change of a few amino acids did not remove or modify either sequential or conformational epitopes (Figure 5 and Tables 3–5).

#### Predicted T-Cell Epitopes of OmpA Protein

NetCTL server of the IEDB analysis resource was used to predict MHC class I T-cell epitopes. We gave the primary protein sequence as input and left the values of 0.15 and 0.05 by default, relative to weights for C-terminal cleavage and TAP transport efficiency, respectively. All serotypes (*n* = 12) were selected for the study. A value of 0.5 was given as an input, allowing the screening of epitopes at a sensitivity of 89% and a specificity of 94%. Among several epitopes predicted by the NetCTL server, we only selected the best five [TCE1(I)–TCE5(I)] based on the high combinatorial score of NetCTL. Table 6 shows MHC class I T-cell epitopes predicted by NetCTL, which are related to the A1, B27, A1, B8, and B58 supertypes, respectively. The epitopes are ordered according to the decreasing order of the NetCTL score.

**Table 6:** The selected best five potential epitopes of the OmpA protein of *Anaplasma marginale* binding to MHC class I alleles, on the basis of their overall score predicted by the NetCTL server, and epitope conservancy result.

**Number**	**Name**	**Start**	**End**	**Peptide sequence**	**Overall**	**Supertypes**	**Epitope conservancy**
		**position**	**position**	**(9-mer)**	**score (nM)**		**(conserved sequences/total)**
1	TCE1(I)	86	95	GTEEYNLAL	1.5946	A1	100% (9/9)
2	TCE2(I)	97	106	RRANAVKEF	1.8235	B27	100% (9/9)
3	TCE3(I)	124	133	KAEPEVLVY	2.0026	A1	100% (9/9)
4	TCE4(I)	143	152	HAQNRRV(F)VL	2.0276	B8	88.9% (8/9)
5	TCE5(I)	165	174	MAIKWPFSF	1.9799	B58	100% (9/9)

*The reference sequence considered in this analysis is that deduced from the genetic variant V1 (GenBank accession number MK882880). The letters found in parentheses represent the amino acids substituted in at least one of the proteins deduced from genetic variants other than the reference variant V1*.

In order to filter the antigenic epitopes from the non-antigenic epitopes, we again used the Kolaskar and Tongaonkar antigenicity prediction tool at the IEDB analysis resource; however, this time, the window length was set to 9. From Kolaskar and Tongaonkar antigenicity prediction (Supplementary Figure 4), we could conclude that all selected epitopes (based on NetCTL score) were antigenic noting that the epitopes TCE2(I), TCE3(I), and TCE4(I) are more antigenic than TCE1(I) and TCE5(I). In addition, all predicted epitopes were 100% conserved in all *A. marginale* isolates except the TCE4(I) epitope, which contains a single mutation at position 149 in a single variant, which did not cause deletion or modification of this epitope (Figure 4 and Table 6).

For predicting the epitopes binding to MHC class II alleles, we used IEDB online server. The server predicts the MHC class II binding regions from the primary amino acid sequence using uses the Consensus approach, combining NN-align, SMM-align, CombLib, and Sturniolo. The primary amino acid sequence of OmpA protein (V1, MK882880) was given as input in FASTA format. Of the several epitopes predicted by the IEDB server, we considered only the top five epitopes [TCE1(II)–TCE5(II)] based on the low adjusted rank score, which means high MHC class II binders. Table 7 lists the predicted MHC class II T-cell epitopes by NetCTL, which are binding to MHC class II alleles HLA-DRB1^*^15:01, HLA-DRB5^*^01:01, HLA-DRB5^*^01:01, HLA-DRB5^*^01:01, and HLA-DRB5^*^01:01. The epitopes are ordered based on the ascending order of the adjusted rank score. To filter antigenic epitopes from non-antigenic epitopes, we again used Kolaskar and Tongaonkar antigenicity prediction tool at IEDB analysis resource, and the window length was set to 9. From Kolaskar and Tongaonkar antigenicity prediction (Supplementary Figure 4), we could conclude that all selected epitopes were antigenic noting that the epitopes TCE1(II), TCE2(II), and TCE4(II) are more antigenic than TCE3(I) and TCE5(I). In addition, all predicted epitopes binding to MHC class II alleles were 100% conserved in all *A. marginale* isolates (Figure 4 and Table 7).

**Table 7:** The selected best five potential epitopes binding to MHC class II alleles, on the basis of their adjusted rank predicted by the IEDB server and epitope conservancy result.

**Number**	**Name**	**Start**	**End**	**Peptide sequence**	**Adjusted rank**	**Allele/haplotype**	**Epitope conservancy**
				**(15-mer)**			**(conserved sequences/total)**
1	TCE1(II)	2	16	LHRWLALCFLASFAV	1.90	HLA-DRB1*15:01	100% (9/9)
2	TCE2(II)	48	62	FNKYEIKGSGKKVLL	1.60	HLA-DRB5*01:01	100% (9/9)
3	TCE3(II)	87	101	TEEYNLALGERRANA	2.40	HLA-DRB5*01:01	100% (9/9)
4	TCE4(II)	150	164	VLIVECQHSVSPKKK	3.60	HLA-DRB5*01:01	100% (9/9)
5	TCE5(II)	167	181	IKWPFSFGRSAAKQD	2.60	HLA-DRB5*01:01	100% (9/9)

*The reference sequence considered in this analysis is that deduced from the genetic variant V1 (GenBank accession number MK882880). The letters found in parentheses represent the amino acids substituted in at least one of the proteins deduced from genetic variants other than the reference variant V1*.

In order to assess the antigenic features of the epitopes binding to MHC class I and II alleles, we predicted their secondary structure using SOPMA Server software. A greater proportion of extended strands and random coils present in the structure of all predicted epitopes except TCE1(I), TCE2(I), and TCE3(II) corresponded with an increased likelihood of these peptides forming antigenic epitopes. The predicted secondary structure results for the potential epitopes binding to MHC class I and II alleles are demonstrated in Figure 5.

## Discussion

The present study is the first molecular–epidemiological report on *A. marginale* infection in cattle covering several geographical and bioclimatic areas extending from the north to the south of Tunisia. Compared with other surveys carried out in Tunisia and other countries, the overall infection rate (2%) estimated in our study is similar to that observed in Turkey (2.3%) (51), Egypt (3.7%) (52), Sudan (6.1%) (53), Kenya (7.8%) (54), and Mongolia (8.7%) (55). However, this rate is lower than that reported in Algeria (11.1%) (56), Pakistan (16.3%) (57), Morocco (21.9%) (58), and other regions from Tunisia (31.5%) (11). Compared with other studies from African countries, *A. marginale* prevalence rates were significantly higher in cattle from Zambia (47.9%) (59), Kenya (31–96.2%) (60, 61), South Africa (60%) (62), Nigeria (75.9%) (63), and Madagascar (89.7%) (64). Indeed, these variations in *A. marginale* prevalence rate between the different countries and regions of the same country could be due to several factors, in particular the sampling seasons and bioclimatic zones, the type of used molecular assays and associated genes, the variation of incriminated arthropod vectors, the susceptibility of animal breeds, and/or other risk factors related to herd management (2, 15).

In this study, a significant discrepancy in *A. marginale* infection prevalence was revealed among bioclimatic areas. In fact, cattle from subhumid area were more infected than those from other bioclimatic areas. This difference, which was also reported in cross-sectional and longitudinal surveys performed on *A. marginale* infection in cattle from Tunisia (11, 15, 16) and Morocco (58), is probably caused by the effect of bioclimatic conditions on the phenology and the distribution of tick vectors and biting flies. Additionally, overall, *A. marginale* prevalence rate differed statistically among geographic regions. This discrepancy may be mainly due to differences in husbandry practices, farm management, tick control programs, and/or wildlife reservoirs (2, 58) but also to bias in sample recruitment. However, in spite of this, our study has confirmed that cattle infested by ticks were statistically more infected by *A. marginale* than those free of ticks. Our results are in agreement with other Tunisian studies that reported that cattle and small ruminants infested with ticks were statistically more infected with *Anaplasma* spp. than those free of ticks (2, 11, 65).

During the last two decades, several methods and markers have been developed to study the phylogeography of different worldwide *A. marginale* isolates. Most of the phylogenetic analyses of *A. marginale* strains were performed using partial sequences of genes that encode merozoite surface proteins (MSPs), mainly *msp4* and *msp1*α (11, 66, 67). However, due to the high degree of sequence diversity in endemic areas, *msp4* gene did not provide phylogeographic information on a global scale, but this gene could be useful in regional-level strain comparisons and could provide important data on host–pathogen coevolution and vector–pathogen relationships (11, 13, 68, 69). On the other hand, *msp1*α partial sequences did not provide a high phylogeographic resolution given that this gene is subjected to a positive selection pressure and appears to be rapidly evolving (67).

Recently, an MLST approach was used in order to gain more complete knowledge about the evolution and the genetic diversity of *A. marginale* strains. This typing method theoretically constitutes an interesting alternative, mainly by its multi-locus power, and offers the advantage that the selection of target loci does not require a complete knowledge of the whole genome but only by using generally seven housekeeping genes sequenced from studied isolates. Thereby, Guillemi et al. (10) developed and applied this method on *A. marginale*; however, by studying these concatenary sequences, they did not find an evident association between the geographic regions and the genetic variants isolated from different isolates from several regions of the world. For this reason, Ben Said et al. (14) examined each of these seven markers independently using the single locus analysis method. Phylogenetic analysis of each locus revealed that out of the seven analyzed genes, two (*lipA* and *sucB*) appear to be more interesting phylogeographically compared with other genes (14).

Therefore, in this study, we choose to assess the phylogeography of our *A. marginale* isolates by the genetic analysis of *lipA* and *sucB* markers. The molecular study based on *lipA* partial sequence revealed that, out of the 14 analyzed isolates, five different genotypes, two of which were novel, were identified (Table 2). This relatively high heterogeneity in relation to the number of infected cattle probably reflects movements of cattle between different countries and regions of the same country. Based on the same gene, similar results were observed by analyzing *A. marginale* isolates from Italy (10) and Tunisia (14).

Phylogenetic analysis based on *lipA* gene allowed the classification of our isolates according to five main clusters strongly supported by bootstrap values ≥75% with some minor exceptions [only six (6.87%) sequences out of 87 are not classified in their appropriate geographic regions]. In particular, this marker clearly differentiates between strains from South African (cluster 1), North American (cluster 2), Mediterranean (cluster 3), Latin American (cluster 4), and North African (cluster 5) countries (Figure 2A). Most of our Tunisian isolates (10/12) were assigned to the third and fifth clusters, confirming the discriminative power of *lipA* gene and a greater heterogeneity of our isolates compared with those found in other countries as previously suggested by Ben Said et al. (14). This finding could be due to the importance of cattle mobility through commercial exchanges between Tunisian regions and with other neighboring countries.

Moreover, *sucB* partial sequence also allows classifying strains according to geographical regions. However, this classification is not according to all or part of continents as for *lipA* gene but according to the New (cluster 1) and the Old World (cluster 2) (Figure 2B) as recently suggested by Ben Said et al. (14). This finding was strongly supported by a high bootstrap value estimated at 82% and the classification of 76 (93.82%) *sucB* partial sequences out of 81 in their appropriate geographic regions (Figure 2B). Otherwise, sequence analysis showed that *sucB* partial sequence was conserved in Tunisian *A. marginale* isolates. Indeed, only one genotype (sucBTunGv1) was revealed in seven sequenced samples. This is in agreement with the results of Guillemi et al. (10), which showed low diversity given that only one genotype was recorded in 39 Latin American isolates. In addition to sucBGv2, sucBGv3, and sucBGv4 genotypes earlier reported by Ben Said et al. (14), a genotype (sucBTunGv1) previously described in Tunisia (sucBGv1) was also detected in seven infected cattle from five different studied regions and, therefore, appears to be predominant in our country (Figure 2B and Table 2).

One of the main limitations to the development of an effective vaccine against *A. marginale* is the diversity of strains but also the inability to produce cross-protection against various isolates with a single vaccine. Therefore, the search for potential vaccine antigen candidates has focused on identifying outer membrane proteins that are widely conserved in remote geographic areas (8). Recently, the protein OmpA, annotated Am854 in the *A. marginale* genome, has been identified as an adhesin playing an essential role in the entry of *A. marginale* bacteria in mammalian and tick cells (9). Therefore, this protein could serve as a highly relevant vaccine antigen candidate (8). In fact, the conservation of the OmpA protein in different regions of the world is essential for the development of an international commercial vaccine. In Tunisia, where potentially pathogenic *A. marginale* strains can be ubiquitous in our farms (14), there is an almost complete lack of information regarding the extent of genetic variation in vaccine candidate proteins like OmpA protein.

In this study, a molecular and phylogenetic study followed by a physicochemical characteristics analysis and a prediction of B- and T-cell epitopes, and secondary and tertiary structures of the deduced OmpA proteins were performed from all available protein variants. Subsequently, an evaluation of the conservation of this protein in its linear, secondary, and tertiary forms and of the predicted epitopes was established between OmpA variants deduced from Tunisian isolates and those published in GenBank.

Genetic analysis of nucleotide and amino acid sequences and phylogenetic study based on *ompA* gene and its deduced protein demonstrated that the OmpA antigen appears to be highly conserved between isolates from geographically diverse worldwide regions. Indeed, a maximum of five amino acid differences were recorded between all OmpA protein variants available in GenBank and the Tunisian variants revealed in this study with amino acid sequence homology >98.2% (Supplementary Tables 5, 6). Our result agrees with that of Futse et al. (8), who demonstrated a high conservation of the OmpA protein in its linear form in Ghanaian *A. marginale* strains by comparing them to the reference variant V1 representing St. Maries, Virginia, Kansas 6DE, Colville C51 and C52, and Nayarit (MX) N3574 strains.

The creation of epitope-based vaccines is a difficult and highly specific technology, requiring the use of molecular biology and immunology techniques. Obtaining the necessary information on the epitopes of the candidate antigen is one of the crucial steps in vaccine design. During this decade, the evolution of bioinformatics tools allowed the improvement of the quality of epitope prediction.

Indeed, many factors influence the vaccine efficacy as the physicochemical parameters, the structure, and the location of protein candidates (70). In addition, the secondary structure of the protein is involved in the distribution of epitopes. Moreover, antigenicity and hydrophilicity are the main factors involved in epitope formation, although interrelated factors, such as flexibility, exposed area, and conformation of secondary and tertiary structure, are also essential.

In the secondary structure of proteins, alpha helices and beta sheets are very regular components, which do not easily deform owing to the presence of hydrogen bonds, which act to maintain a certain structural stability. However, alpha helices and beta sheets do not allow easy ligand binding since they are usually located inside the protein. In contrast, beta-turns and random coil regions are located on the protein surface, thus ensuring the functional needs of the protein. Therefore, these structures are suitable for binding ligands and, consequently, have a high possibility of forming epitopes.

OmpA protein, analyzed by the SOPMA software, potentially consists of 30.51% alpha helix (Hh), 22.03% extended strand (Ee), 7.20% beta-turn (Tt), and 40.25% random coil (Cc). Using this tool, we found that random coil and beta-turn are the most represented types of secondary structure in the OmpA protein. Therefore, we assume that this protein has the required characteristics to be an interesting vaccine candidate. In addition, we predicted the tertiary structures of the OmpA protein from the deduced amino acid sequences of all available genetic variants using I-TASSER, which were refined by ModRefiner server and then validated using the Ramachandran plot, which also confirmed the structural stability of this protein. Therefore, we assume that OmpA protein has the required characteristics to be an interesting vaccine candidate and that the minimal differences found between OmpA amino acid sequences will probably not affect protein binding.

An effective vaccine candidate must not only have a stable protein structure but also be able to induce a powerful immune response. The ideal is to find a vaccine that triggers both humoral and cellular immunity (71). As the combined effects of T and B cells are necessary for antigen removal, it was also important to analyze the T- and B-cell epitopes of the OmpA antigen.

In this study, we identified B-cell epitopes (linear, conformational, and common epitopes) on the basis of several different characteristics such as the antigenicity, the accessibility, the hydrophilicity, the flexibility, and the secondary structure, by using the IEDB, BepiPred, ABCpred, BepiPred 2.0, and CBTope servers. The results of the predicted sites and conformations of B-cell epitopes showed that all predicted epitopes were accessible, flexible, hydrophilic, and found in the beta-turn (Tt) and/or the random coils (Cc) regions (Supplementary Figure 3). In addition, most of these epitopes were antigenic (8/9, 7/8, and 3/4, respectively, for linear, conformational, and common epitopes) (Supplementary Figure 3).

Moreover, T-cell epitopes have to transform into peptides, which bind to the corresponding MHC and are then recognized by the T-cell receptor (TCR) (71). T-cell epitopes are presented by MHC class I and MHC class II molecules, which are recognized by long-term culture (LTc) and T helper (Th) cells, respectively. In this study, the five best selected potential epitopes binding to MHC class I alleles were predicted by the NetCTL server, and the five best selected potential epitopes binding to MHC class II alleles were selected by the IEDB server. The results of the predicted T-cell epitope sites and conformations demonstrated that all of the predicted T-cell epitopes were antigenic (Supplementary Figure 4) and almost were mainly found in the beta-turn (Tt) and/or the random coil (Cc) regions (4/5 for both types of epitopes binding to MHC class I and II alleles) (Figure 4).

After the selection of the epitopes of the B and T cells present in the OmpA protein, we confirmed that the amino acid differences between all available OmpA protein variants so far have neither removed nor modified the epitopes of B and T cells (Figure 4 and Tables 3–7). These results suggest that the minimal differences found between the OmpA sequences will not affect the bindings to B-cell antibodies and to MHC class I and class II T cells when using this antigen in a recombinant or multi-epitope vaccine.

## Conclusion

In this study, the analysis of *lipA* phylogeographic marker indicated a higher diversity of Tunisian *A. marginale* isolates compared with other available worldwide isolates and strains, probably due to multiple introductions from infected cattle from different origins. Despite this heterogeneity, the analysis of the vaccine candidate OmpA protein demonstrated that this antigen appears to be highly conserved, suggesting that the minimal intraspecific modifications will not affect the potential cross-protective capacity of humoral and cell-mediated immune responses against multiple *A. marginale* strains. However, experimental and immunologic studies are needed to confirm this assumption.

## Data Availability Statement

The datasets presented in this study can be found in online repositories. The names of the repository/repositories and accession number(s) can be found in the article/Supplementary Material.

## Ethics Statement

The animal study was reviewed and approved by the Ethics Committee of the National School of Veterinary Medicine of Sidi Thabet, University of Manouba. Written informed consent was obtained from the owners for the participation of their animals in this study.

## Author Contributions

MBS, LM, and HB conceived the idea. MBA, RA, RS, and MM carried out the blood sampling. HB, RA, RS, MK, and MBA performed the experiments. HB, SZ, MBA, RA, and MBS performed risk factor analysis and bioinformatics study. HB and MBS wrote the manuscript. HB, RS, MD, LM, and MBS finalized it. All authors have read and approved the final version.

## Funding

This work was supported by the research laboratory Epidemiology of enzootic infections in herbivores in Tunisia: application to the control (LR16AGR01), and the research projects Screening and molecular characterization of pathogenic and zoonotic bacteria of medical, and economic interest in cattle and camel ticks in Tunisia (19PEJC07-22) and Study of the bacterial microbiota in ticks with a medical and economic impact in Tunisia: contribution to the control of vector-borne bacterial diseases (P2ES2020-D4P1), all of which are funded by the Ministry of Higher Education and Scientific Research of Tunisia.

## Conflict of Interest

The authors declare that the research was conducted in the absence of any commercial or financial relationships that could be construed as a potential conflict of interest.

## Publisher's Note

All claims expressed in this article are solely those of the authors and do not necessarily represent those of their affiliated organizations, or those of the publisher, the editors and the reviewers. Any product that may be evaluated in this article, or claim that may be made by its manufacturer, is not guaranteed or endorsed by the publisher.

## Supplementary Material

Supplementary Figure 1 Nucleotide **(A)** and amino-acid **(B)** alignments showing differences between all *sucB* genetic variants available until this study.

Supplementary Figure 2 Graph representing the best refined model of the OmpA sequence of the 3D structure visualized by PyMol software **(A)** and the Ramachandran plot of the initial model (1) and the best refined model (2) of the candidate developed vaccine **(B)**. The best refined model represents the variant protein OmpATunGv2. The coloring/shading on the Ramachandran plot represents the different regions: the darkest areas (in red) correspond to the “main” regions representing the most favorable combinations of phi-psi values. According to the results, the distribution of the residuals in the refined model was refined by 25.7, 14.7, 9.1, and 2% in the main, authorized, generous, and non-authorized regions, respectively, compared to the initial model.

Supplementary Figure 3 Results of the prediction of the hydrophilicity of Parker **(A)**, the accessibility to Emini surface **(B)**, the flexibility by Karplus and Schulz **(C)**, and the antigenicity of Kolaskar and Tongaonkar **(D)** within the OmpA protein showing the location of the predicted B cell-bound epitopes. The x and y axes represent, respectively, the position of the sequence and the score relating to each analyzed factor. The cut-off values are 2.098, 1.000, 1.005, and 1.030, respectively, for hydrophilicity, accessibility, flexibility, and antigenicity. Regions above the threshold are hydrophilic, contain the “Beta turn” structure type, accessible, flexible, and antigenic, and are shown in yellow. The black lines represent the sequential (SE1–SE8) and conformational (CE1–CE9) epitopes. The rectangles colored in red represent the epitopes selected in common between the sequential and conformational epitopes.

Supplementary Figure 4 Result of the prediction of the antigenicity of Kolaskar and Tongaonkar within the OmpA protein of *A. marginale* showing the location of predicted T cell epitopes. The x and y axes represent, respectively, the position of the sequence and the score for the antigenicity of the protein. The threshold value is 1.030. Regions above the threshold are potentially considered antigenic and are shown in yellow. The rectangles colored in green and orange represent the epitopes [TCE1(I)-TCE5(I)] binding to MHC class I T cells and the epitopes [TCE1(II)–TCE5(II)] binding to MHC class T cells II.

Supplementary Table 1 Single PCR primers used for the identification and/or the genetic characterization of *Anaplasmataceae* and *Anaplasma marginale* infecting cattle from Tunisia.

Supplementary Table 2 Nucleotide **(Bottom)** and amino-acid **(Top)** homology rates between all available genetic variants based on the analyzed *lipA* partial sequence. ^a^Name of the variant, genotype, or isolate. Isolate “LA846” is found in Argentinean cattle and represented by GenBank accession number KM091034. The isolates “Italia7” and “Italia8” are isolated from cattle located in Italy and represented by GenBank accession numbers KM091031 and KM091032, respectively. The strain “St. Maries” is isolated from cattle in USA and represented by GenBank accession number CP000030. The “Africa” isolate is isolated from South African cattle and represented by GenBank accession number KM091016.

Supplementary Table 3 Nucleotidic and amino-acid differences between different *lipA* genetic variants available until this study. ^a^Name of the genetic variant, genotype, or isolate. ^b^The numbers represent the nucleotide positions relative to the *A. marginale lipA* sequence of the St. Maries strain from USA (GenBank accession number CP000030). The conserved nucleotidic positions relative to the first sequence are indicated by asterisks. Amino acid changes, if they exist, are shown in parentheses with a single letter code.

Supplementary Table 4 Nucleotide **(Bottom)** and amino-acid **(Top)** homology rates between all available genetic variants based on the analyzed *sucB* partial sequence. ^a^Name of the genetic variant, genotype, or isolate.

Supplementary Table 5 Nucleotide **(Bottom)** and amino-acid **(Top)** homology rates between all available genetic variants based on the studied *ompA* partial sequence. ^a^Name of the genetic variant or genotype. V1 represents the strain “St. Maries” (GenBank accession number MK882880) infecting cattle in USA and other strains with the same sequence. V2 represents strain “Dawn” isolated from Australian cattle (GenBank accession number KM821232) and other strains with the same sequence. V3 represents the isolate “Emphi” from infected cattle in USA (GenBank accession number KM821235). GV1 represents the isolate “Gha147” (GenBank accession number MK882880) isolated from a Ghanaian cattle and other Ghanaian isolates with the same sequence. GV2 represents the isolate “Gha24” (GenBank accession number MK882857) isolated from a Ghanaian cattle and other Ghanaian isolates with the same sequence. GV3 represents the isolate “Gha50” (GenBank accession number MK882871) isolated from a Ghanaian cattle and other Ghanaian isolates with the same sequence.

Supplementary Table 6 Nucleotide and amino-acid differences between different *ompA* genetic variants available until this study. ^a^Name of the variant, genotype, or isolate. ^b^Numbers represent nucleotide positions relative to *A. marginale ompA* sequence of St. Maries strain from the USA (GenBank accession number CP000030). The conserved nucleotide positions with respect to the first sequence are indicated with asterisks. Positions where sequencing has not been performed are represented by dashes. Amino acid changes are shown in parentheses with a single letter code. Amino-acids: S, serine; L, leucine; V, valine; F, phenylalanine; G, glycine; P, proline; E, glutamic acid; K, lysine. Nucleotides: T, thymine; C, cytosine; G, guanine; A, adenine.
