# Supplementary material for: Screening and Analysis of Anaplasma marginale Tunisian Isolates Reveal the Diversity of lipA Phylogeographic Marker and the Conservation of OmpA Protein Vaccine Candidate
Source: Front Vet Sci. 2021 Oct 21;8:731200. doi: 10.3389/fvets.2021.731200 (PMC8566978; doi:10.3389/fvets.2021.731200)
Supplement: Supplementary Figure 1 — Nucleotide (A) and amino-acid (B) alignments showing differences between all sucB genetic variants available until this study. [file Data_Sheet_1.DOCX]

**(A)**

LH917 ACCAGGGGCCAGGTTTTGGCGATCATCAGCAAGCATGAAGGTGCCCCTCAGGATGCCGCAGCGCGTGAGCACAAGCAAGCTGAAGTTGCAACTCCAGATG 100

Mer_2_May13 ----------------------------------g----------------------------------------------------------------- 100

Florida ---------------------------------------------------------------------------------------------------- 100

Tamaulipas 6 ---------------------------------------------------------------------------------------------------- 100

LA802 ----------------------------------g----------------------------------------------------------------- 100

Oklahoma ---------------------------------------------------------------------------------------------------- 100

LM3 ------------------------------------------------------------------------------------------g--------- 100

COB14 ---------------------------------------------------------------------------------------------------- 100

Italia 6 ---------------------------------------------------------------------------------------------------- 100

Italia 8 ------------------------------------------------------------------------------------------g----g---- 100

Italia 10 ------------------------------------------------------------------------------------------g----g---- 100

LF252 -----------------------------------------------------------------------------------------g---------- 100

Africa ------------------------------------------------------------------------------------------g----g---- 100

sucBGv1 ------------------------------------------------------------------------------------------g----g---- 100

sucBGv2 ---------------------------------------------------------------------------------------------------- 100

sucBGv3 ------------------------------------------------------------------------------------------g----g---- 100

sucBGv4 ------------------------------------------------------------------------------------------g----g---- 100

sucBTunGv1 ------------------------------------------------------------------------------------------g----g---- 100

LH917 CCGAATTAGCACCGCAGGTTGAGCAACGTGATGCGCAAGCTCAAGTTGCTGACAAGGAAAAGCCGGTTAAGCCGGTCACTGGCCCACGCATTCCTGGTAT 200

Mer_2_May13 ---------------------------------------------------------------------------------------------------- 200

Florida ---------------------------------------------------------------------------------------------------- 200

Tamaulipas 6 ---------------------------------------------------------------------------------------------------- 200

LA802 ---------------------------------------------------------------------------------------------------- 200

Oklahoma ---------------------------------------------------------------------------------------------------- 200

LM3 ---------------------------------------------------------------------------------------------------- 200

COB14 ---------------------------------------------------------------------------------------------------- 200

Italia 6 ---------------------------------------------------------------------------------------------------- 200

Italia 8 ---------------------------------t-----t---------------------------------a-c------------------c----- 200

Italia 10 ---------------------------------t-----t---------------------------------a-c------------------c----- 200

LF252 ---------------------------------------------------------------------------------------------------- 200

Africa ---------------------------------t-----t---------------------------------a-c------------------c----- 200

sucBGv1 ---------------------------------t-----t---------------------------------a-c------------------c----- 200

sucBGv2 ---------------------------------------------------------------------------------------------------- 200

sucBGv3 ---------------------------------t-----t---------------------------------a-c------------------c----- 200

sucBGv4 ---------------------------------t-----t---------------------------------a-c------------------c----- 200

sucBTunGv1 ---------------------------------t-----t---------------------------------a-c------------------c----- 200

LH917 AGACGAGTTCGTCGCTGGTGGGTGTTCCTCACCTGCTGATAGGGCGGCTGGTAAAATCACCAAGCCTGTGGGCGATGTGGGCAAGAGCCCCGTCCCGCAG 300

Mer_2_May13 ---------------------------------------------------------------------------------------------------- 300

Florida ---------------c------------------------------------------------------------------------------------ 300

Tamaulipas 6 ---------------c------------------------------------------------------------------------------------ 300

LA802 ---------------------------------------------------------------------------------------------------- 300

Oklahoma ---------------------------------------------------------------------------------------------------- 300

LM3 ---------------------------------------------------------------------------------------------------- 300

COB14 ---------------------------------------------------------------------------------------------------- 300

Italia 6 ---------------------------------------------------------------------------------------------------- 300

Italia 8 ---------------c------------------------------------------------------------------------------------ 300

Italia 10 ---------------c------------------------------------------------------------------------------------ 300

LF252 ---------------------------------------------------------------------------------------------------- 300

Africa ---------------c------------------------------------------------------------------------------------ 300

sucBGv1 ---------------c------------------------------------------------------------------------------------ 300

sucBGv2 ---------------------------------------------------------------------------------------------------- 300

sucBGv3 ---------------c------------------------------------------------------------------------------------ 300

sucBGv4 ---------t-----c------------------------------------------------------------------------------------ 300

sucBTunGv1 ---------------c------------------------------------------------------------------------------------ 300

LH917 CAACGCGTGTATGATGAGGTTGACGGCGTCATAAGCGTGCCAGGAGAAAGGCGCGTTAAGATGAGCAAAATCCGCCAGGTGATCGCGTCACGGCTGAAAG 400

Mer_2_May13 --------------------------------------------------------------------------------------------------g- 400

Florida --------------------------------------------------------------------------------------------------g- 400

Tamaulipas 6 --------------------------------------------------------------------g-----------------------------g- 400

LA802 ---------------------------------------------------------------------------------------------------- 400

Oklahoma ---------------------------------------------------------------------------------------------------- 400

LM3 ---------------------------------------------------------------------------------------------------- 400

COB14 ---------------------------------------------------------------------------------------------------- 400

Italia 6 -----------------------------------------------------------------------------------t---------------- 400

Italia 8 ---------------------------a----------------g-----------------------------------------------------g- 400

Italia 10 --------------------------------------------------------------------g-----------------------------g- 400

LF252 ---------------------------------------------------------------------------------------------------- 400

Africa ---------------------------a----------------g-----------------------------------------------------g- 400

sucBGv1 --------------------------------------------------------------------g-----------------------------g- 400

sucBGv2 ---------------------------------------------------------------------------------------------------- 400

sucBGv3 ---------------------------a----------------g-----------------------g-----------------------------g- 400

sucBGv4 --------------------------------------------------------------------g-----------------------------g- 400

sucBTunGv1 --------------------------------------------------------------------g-----------------------------g- 400

LH917 AATCGCAAAATACCGCAGCCACGCTCAGCACTTTTAATGAGGTGGATATGAGTGCTGTTATGGCGCTCAGGGGGAAATATAAGGAAGGCTTTGAGAAGAA 500

Mer_2_May13 ----------c----------------------------------------------------------------------------------------- 500

Florida ----------c----------------------------------------------------------------------------------------- 500

Tamaulipas 6 ----------c----------------------------------------------------------------------------------------- 500

LA802 ----------c----------------------------------------------------------------------------------------- 500

Oklahoma ---------------------------------------------------------------------------------------------------- 500

LM3 ---------------------------------------------------------------------------------------------------- 500

COB14 ---------------------------------------------------------------------------------------------------- 500

Italia 6 ---------------------------------------------------------------------------------------------------- 500

Italia 8 ----------c----------------------------------------------------------------------------------------- 500

Italia 10 ----------c----------------------------------------------------------------------------------------- 500

LF252 ---------------------------------------------------------------------------------------------------- 500

Africa ----------c--------------t-------------------------------------------------------------------------- 500

sucBGv1 ----------c----------------------------------------------------------------------------------------- 500

sucBGv2 ---------------------------------------------------------------------------------------------------- 500

sucBGv3 ----------c----------------------------------------------------------------------------------------- 500

sucBGv4 ----------c----------------------------------------------------------------------------------------- 500

sucBTunGv1 ----------c----------------------------------------------------------------------------------------- 500

LH917 GTATGAGGTGAAACTGGGGTTCATGTCGTTCTTCATCAGGGCCGTGGTGCTGGCTCTCAGAGAGATACCGGTGATTAATGCTGAGATTTCCGGGGATGAG 600

Mer_2_May13 ---------a----------------------------a------------------------------------------------------------- 600

Florida --------------------------------------a------------------------------------------------------------- 600

Tamaulipas 6 --------------------------------------a------------------------------------------------------------- 600

LA802 --------------------------------------a------------------------------------------------------------- 600

Oklahoma ---------------------------------------------------------------------------------------------------- 600

LM3 ---------------------------------------------------------------------------------------------------- 600

COB14 ---------------------------------------------------------------------------------------------------- 600

Italia 6 ---------------------------------------------------------------------------------------------------- 600

Italia 8 ---------------------------------------------------------------------------------------------------- 600

Italia 10 --------------------------------------a------------------------------------------------------------- 600

LF252 ---------------------------------------------------------------------------------------------------- 600

Africa ---------------------------------------------------------------------------------------------------- 600

sucBGv1 --------------------------------------a------------------------------------------------------------- 600

sucBGv2 ---------------------------------------------------------------------------------------------------- 600

sucBGv3 --------------------------------------a------------------------------------------------------------- 600

sucBGv4 --------------------------------------a------------------------------------------------------------- 600

sucBTunGv1 --------------------------------------a------------------------------------------------------------- 600

LH917 ATAATATACCGCGACTACTGCAACATCGGGGTCGCGGTTGGCGGGGATAAGGGGTTAGTAGTTCCGGTGATACGCGGGGCC 681

Mer_2_May13 -----------------------t--------t-----c---ac------------------------------------- 681

Florida -----------------------t--------t-----c---ac------------------g------------------ 681

Tamaulipas 6 -----------------------t--------t-----c---ac------------------g------------------ 681

LA802 --------------------------------------c---ac------------------------------------- 681

Oklahoma -----------------------t--------t-----c---ac------------------------------------- 681

LM3 ------------------------------------------ac------------------------------------- 681

COB14 -----------t------------------------------ac------------------------------------- 681

Italia 6 -----------t------------------------------ac------------------------------------- 681

Italia 8 -----------t------------------------------ac------------------------------------- 681

Italia 10 -----------t------------------------------ac------------------------------------- 681

LF252 ------------------------------------------ac------------------------------------- 681

Africa -----------t------------------------------ac------------------------------------- 681

sucBGv1 -----------------------t--------t-----c---ac------------------------------------- 681

sucBGv2 ------------------------------------------ac------------------------------------- 681

sucBGv3 -----------------------t--------t-----c---ac------------------------------------- 681

sucBGv4 -----------------------t--------t-----c---ac------------------------------------- 681

sucBTunGv1 -----------------------t--------t-----c---ac------------------------------------- 681

(B)

LH917 TRGQVLAIISKHEGAPQDAAAREHKQAEVATPDAELAPQVEQRDAQAQVADKEKPVKPVTGPRIPGIDEFVAGGCSSPADRAAGKITKPVGDVGKSPVPQ 100

Mer_2_May13 -----------r---------------------------------------------------------------------------------------- 100

Florida ---------------------------------------------------------------------------------------------------- 100

Tamaulipas 6 ---------------------------------------------------------------------------------------------------- 100

La802 -----------r---------------------------------------------------------------------------------------- 100

Oklahoma ---------------------------------------------------------------------------------------------------- 100

LM3 ------------------------------a--------------------------------------------------------------------- 100

CoB14 ---------------------------------------------------------------------------------------------------- 100

Italia 6 ---------------------------------------------------------------------------------------------------- 100

Italia 8 ------------------------------a-------------v-v-----------a----------------------------------------- 100

Italia 10 ------------------------------a-------------v-v-----------a----------------------------------------- 100

LF252 ---------------------------------------------------------------------------------------------------- 100

Africa ------------------------------a-------------v-v-----------a----------------------------------------- 100

sucBGv1 ------------------------------a-------------v-v-----------a----------------------------------------- 100

sucBGv2 ---------------------------------------------------------------------------------------------------- 100

sucBGv3 ------------------------------a-------------v-v-----------a----------------------------------------- 100

sucBGv4 ------------------------------a-------------v-v-----------a----------------------------------------- 100

sucBTunGv1 ------------------------------a-------------v-v-----------a----------------------------------------- 100

LH917 QRVYDEVDGVISVPGERRVKMSKIRQVIASRLKESQNTAATLSTFNEVDMSAVMALRGKYKEGFEKKYEVKLGFMSFFIRAVVLALREIPVINAEISGDE 200

Mer_2_May13 -------------------------------------------------------------------------------k-------------------- 200

Florida -------------------------------------------------------------------------------k-------------------- 200

Tamaulipas 6 -------------------------------------------------------------------------------k-------------------- 200

La802 -------------------------------------------------------------------------------k-------------------- 200

Oklahoma ---------------------------------------------------------------------------------------------------- 200

LM3 ---------------------------------------------------------------------------------------------------- 200

CoB14 ---------------------------------------------------------------------------------------------------- 200

Italia 6 ---------------------------------------------------------------------------------------------------- 200

Italia 8 ---------i------------------------------------------------------------------------------------------ 200

Italia 10 -------------------------------------------------------------------------------k-------------------- 200

LF252 ---------------------------------------------------------------------------------------------------- 200

Africa ---------i------------------------------------------------------------------------------------------ 200

sucBGv1 -------------------------------------------------------------------------------k-------------------- 200

sucBGv2 ---------------------------------------------------------------------------------------------------- 200

sucBGv3 ---------i---------------------------------------------------------------------k-------------------- 200

sucBGv4 -------------------------------------------------------------------------------k-------------------- 200

sucBTunGv1 -------------------------------------------------------------------------------k-------------------- 200

LH917 IIYRDYCNIGVAVGGDKGLVVPVIRGA 227

Mer_2_May13 --------------t------------ 227

Florida --------------t------------ 227

Tamaulipas 6 --------------t------------ 227

La802 --------------t------------ 227

Oklahoma --------------t------------ 227

LM3 --------------t------------ 227

CoB14 --------------t------------ 227

Italia 6 --------------t------------ 227

Italia 8 --------------t------------ 227

Italia 10 --------------t------------ 227

LF252 --------------t------------ 227

Africa --------------t------------ 227

sucBGv1 --------------t------------ 227

sucBGv2 --------------t------------ 227

sucBGv3 --------------t------------ 227

sucBGv4 --------------t------------ 227

sucBTunGv1 --------------t------------ 227

**Figure S1: Nucleotide (A) and amino-acid (B) differences between all *sucB* genetic variants available until this study**
