# Supplementary figures and images for: Screening and Analysis of Anaplasma marginale Tunisian Isolates Reveal the Diversity of lipA Phylogeographic Marker and the Conservation of OmpA Protein Vaccine Candidate
Source: Front Vet Sci. 2021 Oct 21;8:731200. doi: 10.3389/fvets.2021.731200 (PMC8566978; doi:10.3389/fvets.2021.731200)

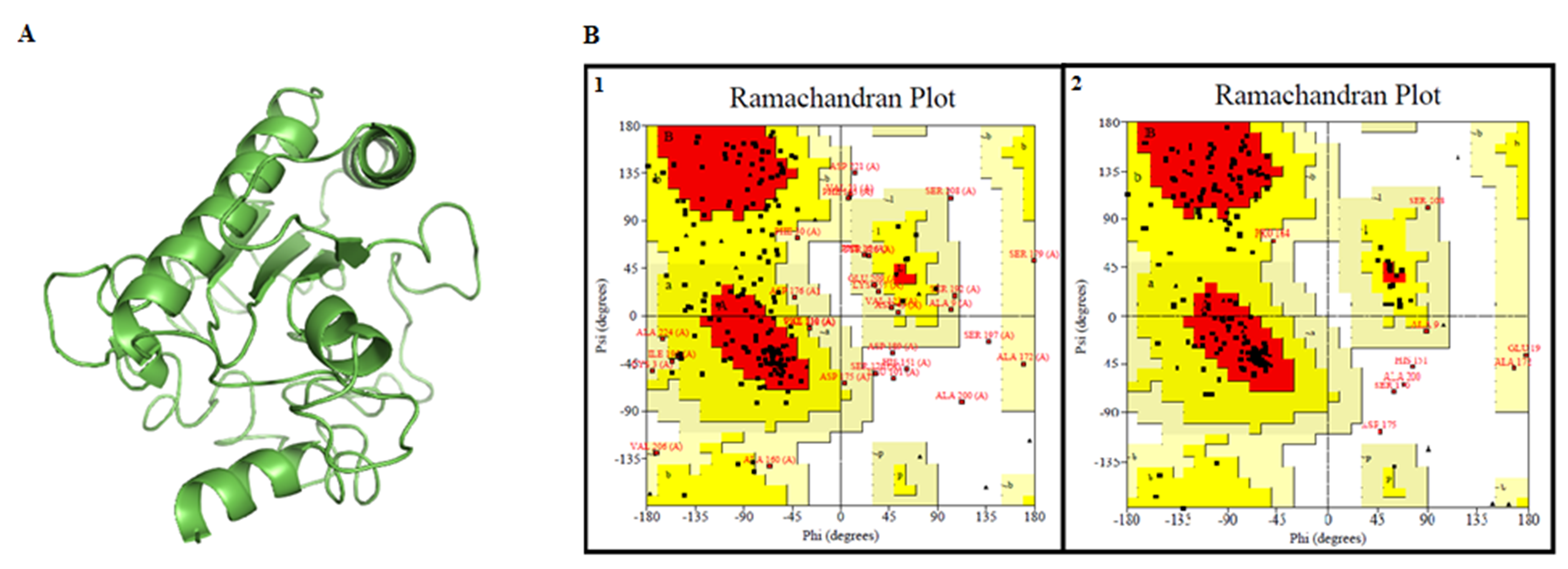

Supplement: Supplementary Figure 2 — Graph representing the best refined model of the OmpA sequence of the 3D structure visualized by PyMol software (A) and the Ramachandran plot of the initial model (1) and the best refined model (2) of the candidate developed vaccine (B). The best refined model represents the variant protein OmpATunGv2. The coloring/shading on the Ramachandran plot represents the different regions: the darkest areas (in red) correspond to the “main” regions representing the most favorable combinations of phi-psi values. According to the results, the distribution of the residuals in the refined model was refined by 25.7, 14.7, 9.1, and 2% in the main, authorized, generous, and non-authorized regions, respectively, compared to the initial model. [file Image_1.TIF]

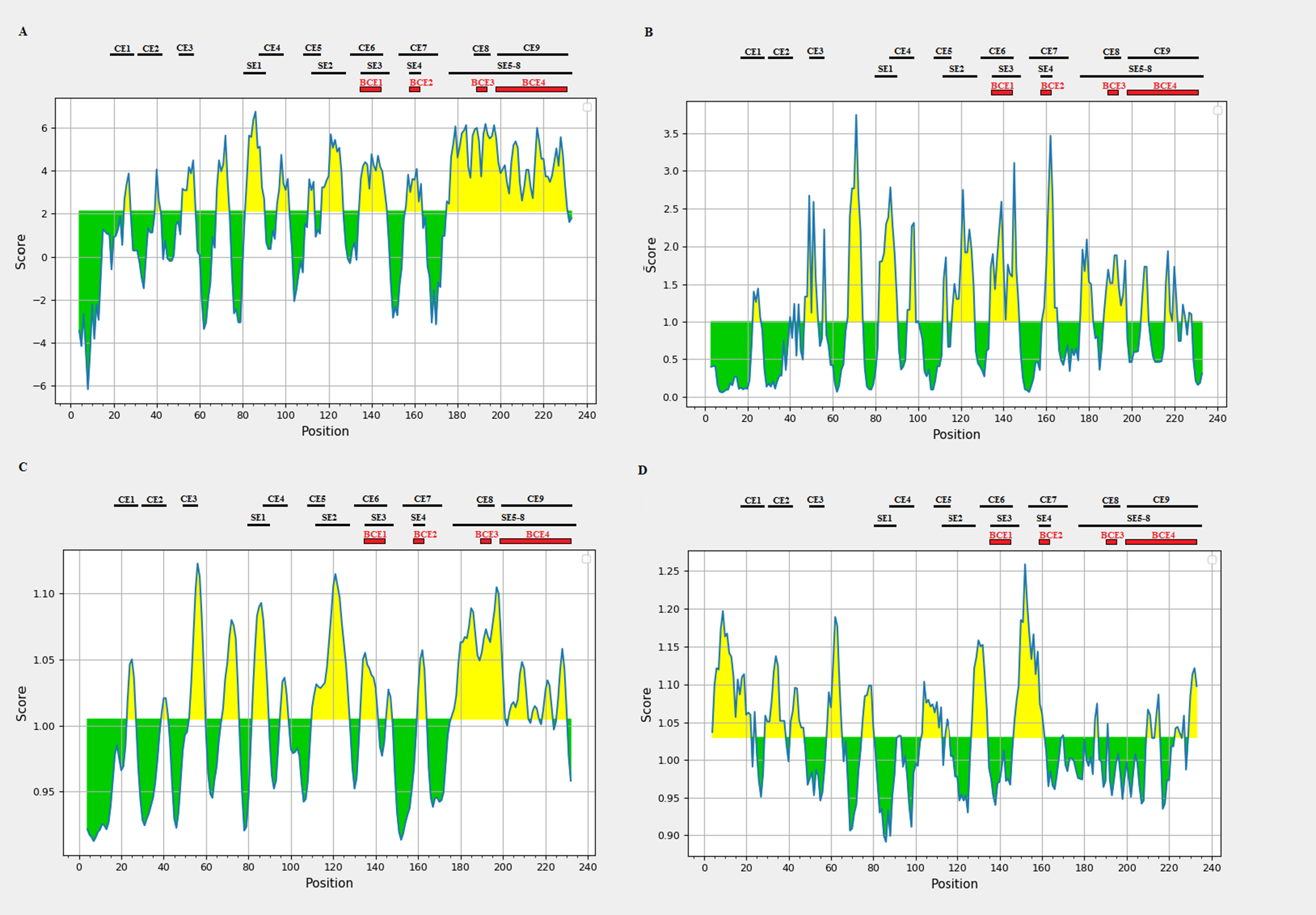

Supplement: Supplementary Figure 3 — Results of the prediction of the hydrophilicity of Parker (A), the accessibility to Emini surface (B), the flexibility by Karplus and Schulz (C), and the antigenicity of Kolaskar and Tongaonkar (D) within the OmpA protein showing the location of the predicted B cell-bound epitopes. The x and y axes represent, respectively, the position of the sequence and the score relating to each analyzed factor. The cut-off values are 2.098, 1.000, 1.005, and 1.030, respectively, for hydrophilicity, accessibility, flexibility, and antigenicity. Regions above the threshold are hydrophilic, contain the “Beta turn” structure type, accessible, flexible, and antigenic, and are shown in yellow. The black lines represent the sequential (SE1–SE8) and conformational (CE1–CE9) epitopes. The rectangles colored in red represent the epitopes selected in common between the sequential and conformational epitopes. [file Image_2.TIF]

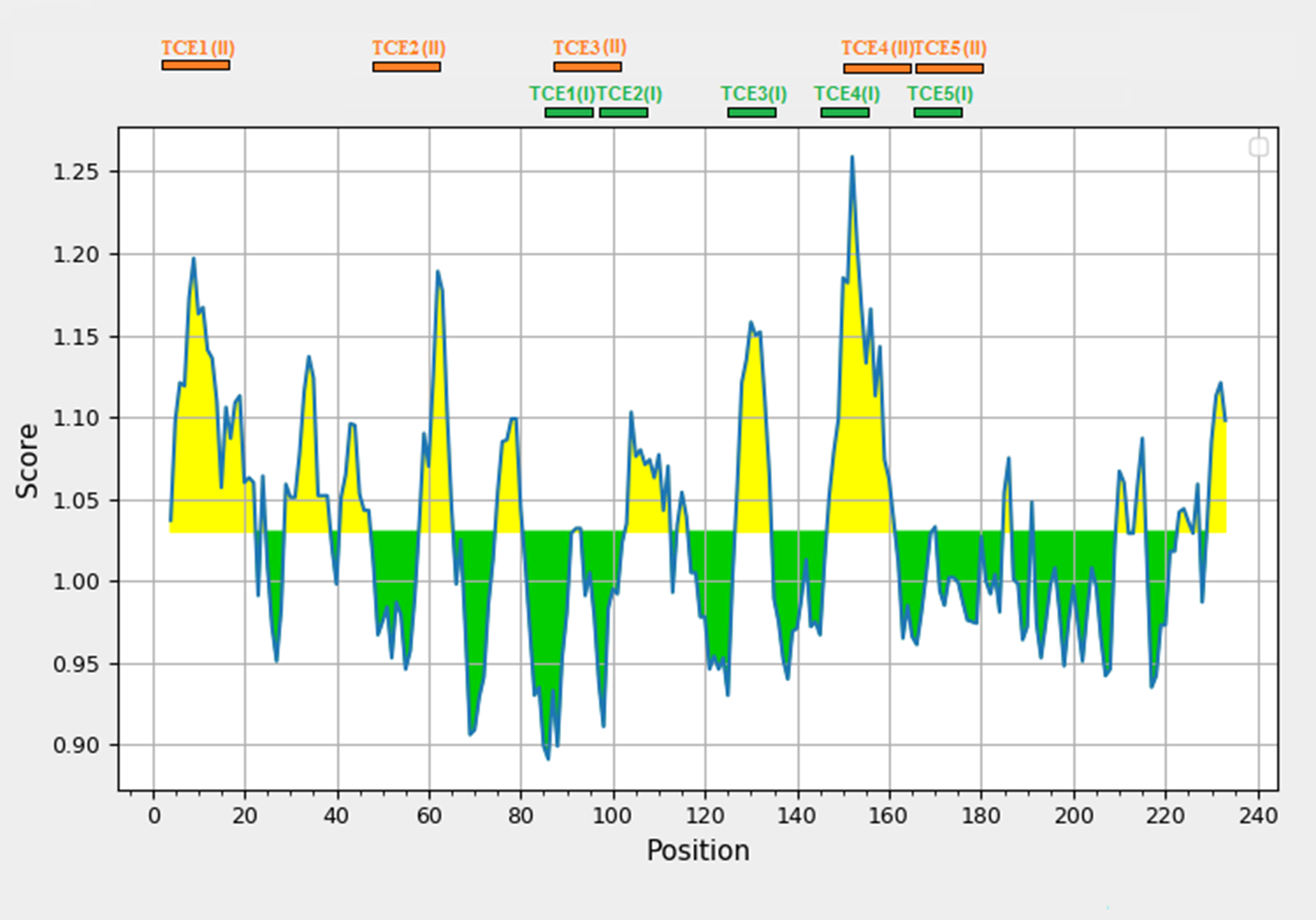

Supplement: Supplementary Figure 4 — Result of the prediction of the antigenicity of Kolaskar and Tongaonkar within the OmpA protein of A. marginale showing the location of predicted T cell epitopes. The x and y axes represent, respectively, the position of the sequence and the score for the antigenicity of the protein. The threshold value is 1.030. Regions above the threshold are potentially considered antigenic and are shown in yellow. The rectangles colored in green and orange represent the epitopes [TCE1(I)-TCE5(I)] binding to MHC class I T cells and the epitopes [TCE1(II)–TCE5(II)] binding to MHC class T cells II. [file Image_3.TIF]
