# Supplementary material for: Screening and Analysis of Anaplasma marginale Tunisian Isolates Reveal the Diversity of lipA Phylogeographic Marker and the Conservation of OmpA Protein Vaccine Candidate
Source: Front Vet Sci. 2021 Oct 21;8:731200. doi: 10.3389/fvets.2021.731200 (PMC8566978; doi:10.3389/fvets.2021.731200)
Supplement: Supplementary Table 1 — Single PCR primers used for the identification and/or the genetic characterization of Anaplasmataceae and Anaplasma marginale infecting cattle from Tunisia. [file Table_1.DOCX]

**Table S1 |** Single PCR primers used for the identification and/or the genetic characterization of *Anaplasmataceae* and *Anaplasma marginale* infecting cattle from Tunisia.

| **Target bacteria** | **Target gene** | **Primer** | **Sequence (5’-3’)** | **Amplicon size (bp)** | **Reference** |
| --- | --- | --- | --- | --- | --- |
| *Anaplasmataceae* | 16S rRNA | EHR 16S D | GGTACCYACAGAAGAAGTCC | 345 | (17) |
|  |  | EHR 16S R | TAGCACTCATCGTTTACAGC |  |  |
| *A. marginale* | *msp4* | Amargmsp4F | CTGAAGGGGGAGTAATGGG | 344 | (18) |
|  |  | Amargmsp4R | GGTAATAGCTGCCAGAGATTCC |  |  |
|  | *sucB* | SucB F | GAGATAGCATCTCCGGTTGC | 808 | (10) |
|  |  | SucB R | CTCCCCTGGCCTTTTTACTC |  |  |
|  | *lipA* | LipA F | TGTGGATAGGGACGACCTTC | 538 | (10) |
|  |  | LipA R | AAAGTCATCCTCAGCGTGGT |  |  |
|  | *ompA* | AmOmpAF | ATGCTGCATCGTTGGTTAGC | 711 | (8) |
|  |  | AmOmpAR | TTCAGGCGCGACCACTCCTG |  |  |

**Table S2 |** Nucleotide (bottom) and amino-acid (top) homology rates between all available genetic variants based on the analyzed *lipA* partial sequence.

| Variant^1^ | Homology rates (%) | | | | | | | | |  |  |
| --- | --- | --- | --- | --- | --- | --- | --- | --- | --- | --- | --- |
|  | 1 | 2 | 3 | 4 | 5 | 6 | 7 | 8 | 9 | 10 | 11 |
| LA846 | 100 | 100 | 100 | 100 | 100 | 100 | 100 | 100 | 100 | 100 | 100 |
| Italia8 | 99.8 | 100 | 100 | 100 | 100 | 100 | 100 | 100 | 100 | 100 | 100 |
| Italia7 | 99.6 | 99.8 | 100 | 100 | 100 | 100 | 100 | 100 | 100 | 100 | 100 |
| St. Maries | 99.2 | 99.0 | 98.8 | 100 | 100 | 100 | 100 | 100 | 100 | 100 | 100 |
| Africa | 99.0 | 99.2 | 99.4 | 99.4 | 100 | 100 | 100 | 100 | 100 | 100 | 100 |
| lipAGv4 | 98.8 | 99.0 | 99.2 | 99.6 | 99.8 | 100 | 100 | 100 | 100 | 100 | 100 |
| lipATunGv1 | 98.8 | 99.0 | 99.2 | 99.6 | 99.8 | 100 | 100 | 100 | 100 | 100 | 100 |
| lipATunGv2 | 98.6 | 98.8 | 99.0 | 99.4 | 99.6 | 99.8 | 99.8 | 100 | 100 | 100 | 100 |
| lipATunGv3 | 98.4 | 98.6 | 98.8 | 99.2 | 99.4 | 99.6 | 99.6 | 99.4 | 100 | 100 | 100 |
| lipATunGv4 | 99.8 | 100 | 99.8 | 99.0 | 99.2 | 99.0 | 99.0 | 98.8 | 98.6 | 100 | 100 |
| lipATunGv5 | 100 | 99.8 | 99.6 | 99.2 | 99.0 | 98.8 | 98.8 | 98.6 | 98.4 | 99.8 | 100 |

*1: Name of the variant, genotype or isolate.*

*Note: Isolate “LA846” is found in Argentinean cattle and represented by GenBank accession number KM091034. The isolates “Italia7” and “Italia8” are isolated from cattle located in Italy and represented by GenBank accession numbers KM091031 and KM091032, respectively. The strain “St. Maries” is isolated from cattle in USA and represented by GenBank accession number CP000030. The “Africa” isolate is isolated from South African cattle and represented by GenBank accession number KM091016.*

**Table S3 |** Nucleotidic and amino-acid differences between different *lipA* genetic variants available until this study.

| **Variant ^1^** | **Nucleotidic positions (amino-acid positions)^2^** | | | | | | | | |
| --- | --- | --- | --- | --- | --- | --- | --- | --- | --- |
|  | 402 | 420 | 438 | 495 | 606 | 639 | 699 | 708 | 726 |
| LA846 | T | A | T | T | A | C | C | A | C |
| Italia8 | * | * | * | C | * | * | * | * | * |
| Italia7 | * | * | C | C | * | * | * | * | * |
| St. Maries | * | * | * | * | G | * | T | G | T |
| Africa | * | * | C | C | * | * | T | G | T |
| lipAGv4 | * | * | C | C | G | * | T | G | T |
| lipATunGv1 | * | * | C | C | G | * | T | G | T |
| lipATunGv2 | * | * | C | C | G | T | T | G | T |
| lipATunGv3 | C | T | C | C | G | * | T | G | T |
| lipATunGv4 | * | * | * | C | * | * | * | * | * |
| lipATunGv5 | * | * | * | * | * | * | * | * | * |

*^1^: Name of the genetic variant, genotype or isolate.*

*^2^: The numbers represent the nucleotide positions relative to the A. marginale lipA sequence of the St. Maries strain from USA (GenBank accession number CP000030). The conserved nucleotidic positions relative to the first sequence are indicated by asterisks. Amino acid changes, if they exist, are shown in parentheses with a single letter code.*

**Table S4 |** Nucleotide (bottom) and amino-acid (top) homology rates between all available genetic variants based on the analyzed *sucB* partial sequence.

| **Variant^1^** | **Homology rates (%)** | | | | | | | | | | | | | | | | | |
| --- | --- | --- | --- | --- | --- | --- | --- | --- | --- | --- | --- | --- | --- | --- | --- | --- | --- | --- |
|  | 1 | 2 | 3 | 4 | 5 | 6 | 7 | 8 | 9 | 10 | 11 | 12 | 13 | 14 | 15 | 16 | 17 | 18 |
| LH917 | 100 | 98.7 | 99.1 | 99.1 | 98.7 | 99.6 | 99.1 | 99.6 | 99.6 | 97.4 | 97.4 | 99.6 | 97.4 | 97.4 | 99.6 | 96.9 | 97.4 | 97.4 |
| Mer_2_may13 | 98.5 | 100 | 99.6 | 99.6 | 100.0 | 99.1 | 98.7 | 99.1 | 99.1 | 96.9 | 97.8 | 99.1 | 96.9 | 97.8 | 99.1 | 97.4 | 97.8 | 97.8 |
| Florida | 98.5 | 99.4 | 100 | 100.0 | 99.6 | 99.6 | 99.1 | 99.6 | 99.6 | 97.4 | 98.2 | 99.6 | 97.4 | 98.2 | 99.6 | 97.8 | 98.2 | 98.2 |
| Tamaulipas 6 | 98.4 | 99.3 | 99.9 | 100 | 99.6 | 99.6 | 99.1 | 99.6 | 99.6 | 97.4 | 98.2 | 99.6 | 97.4 | 98.2 | 99.6 | 97.8 | 98.2 | 98.2 |
| LA802 | 99.1 | 99.4 | 99.1 | 99.0 | 100 | 99.1 | 98.7 | 99.1 | 99.1 | 96.9 | 97.8 | 99.1 | 96.9 | 97.8 | 99.1 | 97.4 | 97.8 | 97.8 |
| Oklahoma | 99.3 | 99.3 | 99.3 | 99.1 | 99.3 | 100 | 99.6 | 100.0 | 100.0 | 97.8 | 97.8 | 100.0 | 97.8 | 97.8 | 100 | 97.4 | 97.8 | 97.8 |
| LM3 | 99.6 | 98.7 | 98.7 | 98.5 | 99.3 | 99.4 | 100 | 99.6 | 99.6 | 98.2 | 98.2 | 99.6 | 98.2 | 98.2 | 99.6 | 97.8 | 98.2 | 98.2 |
| COB14 | 99.6 | 98.7 | 98.7 | 98.5 | 99.3 | 99.4 | 99.7 | 100 | 100.0 | 97.8 | 97.8 | 100.0 | 97.8 | 97.8 | 100.0 | 97.4 | 97.8 | 97.8 |
| Italia 6 | 99.4 | 98.5 | 98.5 | 98.4 | 99.1 | 99.3 | 99.6 | 99.9 | 100 | 97.8 | 97.8 | 100.0 | 97.8 | 97.8 | 100.0 | 97.4 | 97.8 | 97.8 |
| Italia 8 | 97.8 | 97.5 | 97.8 | 97.7 | 97.8 | 97.7 | 98.2 | 98.2 | 98.1 | 100 | 99.1 | 97.8 | 100.0 | 99.1 | 97.8 | 99.6 | 99.1 | 99.1 |
| Italia 10 | 97.8 | 97.8 | 98.1 | 98.2 | 98.1 | 97.7 | 98.2 | 98.2 | 98.1 | 99.4 | 100 | 97.8 | 99.1 | 100.0 | 97.8 | 99.6 | 100.0 | 100.0 |
| LF252 | 99.6 | 98.7 | 98.7 | 98.5 | 99.3 | 99.4 | 99.7 | 99.7 | 99.6 | 97.9 | 97.9 | 100 | 97.8 | 97.8 | 100.0 | 97.4 | 97.8 | 97.8 |
| Africa | 97.7 | 97.4 | 97.7 | 97.5 | 97.7 | 97.5 | 98.1 | 98.1 | 97.9 | 99.9 | 99.3 | 97.8 | 100 | 99.1 | 97.8 | 99.6 | 99.1 | 99.1 |
| sucbgv1 | 97.5 | 98.4 | 98.7 | 98.8 | 98.1 | 98.2 | 97.9 | 97.7 | 97.5 | 98.8 | 99.4 | 97.7 | 98.7 | 100 | 97.8 | 99.6 | 100.0 | 100.0 |
| sucbgv2 | 99.7 | 98.8 | 98.8 | 98.7 | 99.4 | 99.6 | 99.9 | 99.9 | 99.7 | 98.1 | 98.1 | 99.9 | 97.9 | 97.8 | 100 | 97.4 | 97.8 | 97.8 |
| sucbgv3 | 97.2 | 98.1 | 98.4 | 98.5 | 97.8 | 97.9 | 97.7 | 97.4 | 97.2 | 99.1 | 99.1 | 97.4 | 99.0 | 99.7 | 97.5 | 100 | 99.6 | 99.6 |
| sucbgv4 | 97.4 | 98.2 | 98.5 | 98.7 | 97.9 | 98.1 | 97.8 | 97.5 | 97.4 | 98.7 | 99.3 | 97.5 | 98.5 | 99.9 | 97.7 | 99.6 | 100 | 100.0 |
| sucBTunGv1 | 97.5 | 98.4 | 98.7 | 98.8 | 98.1 | 98.2 | 97.9 | 97.7 | 97.5 | 98.8 | 99.4 | 97.7 | 98.7 | 100.0 | 97.8 | 99.7 | 99.9 | 100 |

*^1^: Name of the genetic variant, genotype or isolate.*

**Table S5 |** Nucleotide (bottom) and amino-acid (top) homology rates between all available genetic variants based on the studied *ompA* partial sequence**.**

| Variant^1^ | Homology rates (%) | | | | | | | | |
| --- | --- | --- | --- | --- | --- | --- | --- | --- | --- |
|  | 1 | 2 | 3 | 4 | 5 | 6 | 7 | 8 | 9 |
| V1 | 100 | 100 | 99.6 | 100 | 99.6 | 98.7 | 99.6 | 100 | 99.6 |
| V2 | 99.9 | 100 | 99.6 | 100 | 99.6 | 98.7 | 99.6 | 100 | 99.6 |
| V3 | 99.7 | 99.6 | 100 | 99.6 | 100 | 99.1 | 100 | 99.6 | 99.1 |
| V4 | 99.9 | 99.7 | 99.6 | 100 | 99.6 | 98.7 | 99.6 | 100 | 99.6 |
| GV1 | 99.6 | 99.4 | 99.9 | 99.4 | 100 | 99.1 | 100 | 99.6 | 99.1 |
| GV2 | 99.3 | 99.1 | 99.6 | 99.1 | 99.7 | 100 | 99.1 | 98.7 | 98.2 |
| GV3 | 99.6 | 99.4 | 99.9 | 99.4 | 100 | 99.7 | 100 | 99.6 | 99.1 |
| OmpATunGV1 | 100 | 99.9 | 99.7 | 99.9 | 99.6 | 99.3 | 99.6 | 100 | 99.6 |
| OmpATunGV2 | 99.9 | 99.7 | 99.6 | 99.7 | 99.4 | 99.1 | 99.4 | 99.9 | 100 |

*1: Name of the genetic variant or genotype.*

*Note: V1 represents the strain “St. Maries” (GenBank accession number MK882880) infecting cattle in USA and other strains with the same sequence. V2 represents strain “Dawn” isolated from Australian cattle (GenBank accession number KM821232) and other strains with the same sequence. V3 represents the isolate “Emphi” from infected cattle in USA (GenBank accession number KM821235). GV1 represents the isolate “Gha147” (GenBank accession number MK882880) isolated from a Ghanaian cattle and other Ghanaian isolates with the same sequence. GV2 represents the isolate “Gha24” (GenBank accession number MK882857) isolated from a Ghanaian cattle and other Ghanaian isolates with the same sequence. GV3 represents the isolate “Gha50” (GenBank accession number MK882871) isolated from a Ghanaian cattle and other Ghanaian isolates with the same sequence.*

**Table S6 |** Nucleotide and amino-acid differences between different *ompA* genetic variants available until this study.

| Variant^1^ | Nucleotide positions (amino-acid positions)^2^ | | | | | | | | |
| --- | --- | --- | --- | --- | --- | --- | --- | --- | --- |
|  | 309 | 335 (112) | 363 | 445 (149) | 531 | 556 (186) | 663 | 685 (229) | 706 (236) |
| V1 | G | C (S) | C | G (V) | G | A (S) | G | T (S) | G (E) |
| V2 | * | * | * | * | A | * | * | * | * |
| V3 | * | * | * | * | * | * | A | C (P) | * |
| V4 | A | * | * | * | * | * | * | * | * |
| GV1 | * | * | T | * | * | * | A | C (P) | * |
| GV2 | * | T (L) | T | * | * | G (G) | A | C (P) | * |
| GV3 | * | * | T | * | * | * | A | C (P) | A (K) |
| OmpATunGV1 | * | * | * | * | * | * | * | * | - |
| OmpATunGV2 | * | * | * | T (F) | * | * | * | * | - |

*1: Name of the variant, genotype or isolate.*

*2: Numbers represent nucleotide positions relative to A. marginale ompA sequence of St. Maries strain from the USA (GenBank accession number CP000030). The conserved nucleotide positions with respect to the first sequence are indicated with asterisks. Positions where sequencing has not been performed are represented by dashes. Amino acid changes are shown in parentheses with a single letter code. Amino-acids: S, Serine; L, Leucine; V, Valine; F, Phenylalanine; G, Glycine; P, Proline; E, Glutamic acid; K, Lysine. Nucleotides: T, Thymine; C, Cytosine; G, Guanine; A, Adenine.*
